# Exploring the Genetic Causes of Nonsyndromic Retinal Dystrophies in Qatar

**DOI:** 10.3390/genes16121415

**Published:** 2025-11-27

**Authors:** Sumaya Abiib, Houssein Khodjet-El-khil, Reem Ibrahim Bux, Karen El-Akouri, Sarah Okashah, Tawfeg Ben Omran, Rehab Al Saleh, Mashael Al-Shafai

**Affiliations:** 1 Department of Biomedical Sciences, College of Health Sciences, QU Health, Qatar University, Doha 2713, Qatar; sa1602488@qu.edu.qa (S.A.);; 2 Department of Adult and Pediatric Medical Genetics, Hamad Medical Corporation, Doha 3050, Qatar; 3 Division of Genetic and Genomic Medicine, Sidra Medicine, Doha 26999, Qatar; 4 Biomedical Research Center, Qatar University, Doha 2713, Qatar

**Keywords:** nonsyndromic retinal dystrophy, diagnostic yield, whole exome sequencing, *ABCA4* gene, Qatar

## Abstract

Non-syndromic Inherited Retinal Dystrophies (IRDs) are a set of degenerative retinal diseases that vary clinically and genetically, including Leber congenital amaurosis (LCA) and retinitis pigmentosa (RP). IRDs are a significant cause of vision loss in young adults globally. To date, more than 280 genes have been associated with IRD pathogenesis. This study aims to investigate the genetic basis of non-syndromic IRD in the Qatari population and to assess the diagnostic yield of various genetic tests through a retrospective cohort study. Our study identified 49 eligible patients with IRD, 61.2% of whom were Qatari. Rod-dominated phenotypes accounted for 51% of the hereditary retinal diseases in this cohort. Whole-exome sequencing with mitochondrial genome testing (WES Plus) was the most frequently utilized genetic test. A total of 55 variants were identified across 32 IRD-associated genes. Of the 49 cases, 34 (69.4%) were initially classified as solved, and an additional five were likely to be solved based on familial segregation analysis. Variants in the ABCA4 gene were the most commonly observed, present in eight patients, with the c.5882G>A variant being the most recurrent, identified in three of these cases. Specific genes exhibited recurrent variations, including pan-ethnic variants that are common across multiple populations. These variants merit prioritization in testing due to their global prevalence. WES is recommended as a first-tier test for non-syndromic IRD cases, as it accelerates diagnosis, facilitates earlier interventions, and provides a comprehensive genetic picture by incorporating information from family members. Moreover, our study highlighted the significance of performing family segregation analyses in identifying possible causative variants. This is the first genetic study of IRD in Qatar, laying the groundwork for further research on the epidemiology and genetics of non-syndromic IRD in this understudied region.

## 1. Introduction

Inherited Retinal Dystrophies (IRDs) are a diverse group of genetic conditions that cause severe vision loss by affecting the retina’s structure and/or function [1]. Common manifestations of IRDs include night blindness, color blindness, central or peripheral vision impairments, difficulty with light adaptation, loss of contrast sensitivity, and tunnel vision. In progressive cases, these symptoms can lead to complete blindness [1]. Most IRD cases are non-syndromic, affecting only the retina. In syndromic forms of IRD, other organs and tissues, such as the cardiovascular system, ears, kidneys, and central nervous system, are also affected [2]. Inherited retinal diseases significantly contribute to vision loss in young adults worldwide, with an estimated prevalence of up to 1 in 2000 [3].

IRD comprises several overlapping disorders, including Leber congenital amaurosis (LCA), Stargardt disease, and retinitis pigmentosa (RP) [4]. IRD can be inherited in various patterns, including autosomal dominant (AD), autosomal recessive (AR), mitochondrial, X-linked (XL), or sporadic forms [5]. IRDs are genetically heterogeneous, with more than 250 genes linked to IRD that encode proteins expressed at different levels in the retina [5].

IRDs are classified based on the photoreceptor cells involved and the extent of retinal atrophy [6]. Rod and cone photoreceptors are key cellular units that convert light energy into neuronal signals, enabling visual processing in the brain [6]. There are three types of non-syndromic IRD: generalized retinal degenerations affecting both rod and cone photoreceptors; rod-dominated diseases; and cone-dominated diseases [7].

Implementing advanced molecular techniques such as next-generation sequencing (NGS) has raised the possibility of detecting causative variants in patients with IRD [8]. Identifying causative variants can significantly enhance medical care by offering a prognosis, reducing the need for additional electrophysiological evaluations, and guiding treatment modifications [9]. Genetic testing also enables the proper identification of inheritance patterns, further enhancing genetic counseling services for affected patients and their families [9].

IRD presents a significant challenge for molecular diagnosis due to the large number of genes involved, variable expression, frequent clinical and genetic overlap, and incomplete penetrance [7]. Over the past couple of years, efforts have been concentrated on using the NGS technique to identify variants in IRD [10]. Several organizations have decided to build custom gene panels that sequence a specified list of disease genes [10]. For routine genetic diagnosis, targeted gene panel sequencing provides a rapid, accurate, and relatively cost-effective method for genotype screening. Although fewer genes are evaluated in gene panels compared to whole-exome sequencing (WES), and additional novel genes may not be discovered, the variant detection rate in gene panels can still increase significantly with a proper approach and sufficient depth of coverage [11]. However, since new disease genes in IRD are being discovered at an increasingly rapid rate, gene panels are limited in their ability to include more disease genes as they are identified. Thus, WES has evolved as a fundamental methodological tool that also aids in the discovery of new IRD genes and increases the number of cases that have been successfully diagnosed [11].

The precise incidence of IRDs in Qatar remains unknown. In a study conducted at the Al Noor Institute for the Blind in Qatar, 90 children participated in research to identify the causes and degree of vision loss [12]. Children with consanguineous parents (67.7%) had a significantly higher frequency of visual impairment among family members and were predominantly affected by congenital and/or inherited ocular diseases. This suggests that consanguinity is a significant risk factor for inherited or congenital visual impairment in Qatar [12]. A comprehensive analysis of the genetics of IRDs conducted across Arab nations was published by Jaffal et al. [13]. Thirty-one papers, covering 407 individuals from 11 Arab countries, were reviewed [13]. NGS emerged as the predominant technology in 68% of cases [12]. AR recessive inheritance was identified as the most prevalent inheritance pattern, accounting for 97% of IRD cases. In Saudi Arabia, *RP1* (20%) and *TULP1* (20%) gene variants were frequently observed in patients with retinitis pigmentosa (RP). Conversely, Northern Africa exhibited a higher prevalence of *MERTK* (18%) and *RLBP1* gene variants (18%) [13]. Currently, there is no cure for most types of IRD due to the difficulty of regenerating the affected retinal cells [14]. Nevertheless, with the officially FDA-approved gene therapy for biallelic *RPE65* mutation-associated retinal dystrophy, Luxturna, validating a genetic diagnosis through genetic testing may enable patients to learn about the latest treatment options or qualify them for research participation [15].

This study aims to examine the genetic causes of non-syndromic IRDs within the population of Qatar and to evaluate the diagnostic yield of various genetic tests available. This will provide a better understanding of the molecular spectrum of IRD in the population of Qatar and will assist in providing proper genetic counseling services and options to patients and families with or suspected of having IRD.

## 2. Materials and Methods

### 2.1. Study Design and Participants

Ethical approval for the study was granted by Hamad Medical Corporation (HMC) Medical Research Center (MRC-01-22-729) and by the Institutional Review Board (IRB) of Qatar University (QU-IRB 1803-E/23) following the Declaration of Helsinki. A retrospective chart review of patients’ records seen at the Department of Adult and Pediatric Medical Genetics at Hamad Medical Corporation between 2015 and 2022 was conducted. The approach we used to search for relevant study participants involved employing keywords, including disease names, the names of IRD genes, and/or clinical manifestations of IRD. We reviewed the patients’ records, and the inclusion criteria included patients of both genders and any age with a clinical diagnosis of non-syndromic retinal dystrophy who underwent at least one genetic test. Patients were evaluated by experienced ophthalmologists and clinical geneticists and underwent a comprehensive ophthalmological assessment to confirm the presence and pattern of retinal dystrophy.

To exclude syndromic presentations, systemic and neurological examinations were conducted, supported by review of the patients’ medical history and relevant laboratory findings. Patients showing extra-ocular features suggestive of syndromic conditions (e.g., renal, hepatic, or neurological involvement) were excluded from the cohort. The non-syndromic classification was thus based on clinical findings in conjunction with genetic results. The exclusion criteria included patients with syndromic retinal dystrophies and patients who did not undergo any genetic testing. All eligible patients were given a representative numerical code, and their sociodemographic data were collected. This includes patients’ gender, age, age at diagnosis, nationality, consanguinity, family history, and clinical manifestations.

### 2.2. Genetic Testing

The genetic testing approach for IRD cases in the Department of Adult and Pediatric Medical Genetics at HMC includes IRD gene panels, WES, WES Plus (WES with mitochondrial genome testing), and familial targeted testing for specific gene variants. These tests are conducted in specialized laboratories abroad, following the guidelines of the American College of Medical Genetics and Genomics (ACMG) (https://www.acmg.net/ accessed on 6 July 2024) and the American Association for Molecular Pathology (AMP) classification criteria (https://www.amp.org/ accessed on 6 July 2024). This classification process considers population, computational, functional, and segregation data (https://www.genedx.com/ accessed on 13 May 2024). Patients with an unknown genetic cause are offered WES, WES Plus, or gene panel testing. Patients receiving a negative or inconclusive panel result are subsequently offered WES or WES Plus for more comprehensive testing. If a known IRD familial pathogenic variant is identified, targeted familial testing is provided to family members to detect the specific gene variant associated with the condition. Different gene panels are used in cases of IRDs, including the Congenital Stationary Night Blindness (CSNB) Panel, which tests for 12 genes (Supplemental Table S1). Another available gene panel is the Cone-Rod Dystrophies Panel, which utilizes sequencing analysis to test for 31 genes. The third panel is the Retinal Dystrophy Xpanded Panel, which also utilizes sequencing analysis and can employ a “trio” approach that combines the simultaneous investigation of the affected proband and both parents, thereby increasing the likelihood of identifying a definitive genetic cause in IRD cases. This panel tests for about 780 genes. To ensure accuracy and reliability, all test results conducted by the lab are confirmed through Sanger sequencing. We reviewed all patients’ genetic test results, considering the type of genetic testing performed (familial targeted testing, gene panel testing, WES, or WES Plus), the identified variants and their type, variant classification, associated genes, zygosity status, inheritance pattern, associated phenotypes, and related IRD conditions.

### 2.3. Patient Classification

Patients were classified into three categories—solved, unsolved, and uncertain—based on their genetic findings and the extent to which the identified variants, according to updated ACMG guidelines, accounted for the clinical phenotype. Solved cases were those in which pathogenic or likely pathogenic variants were identified in IRD-associated genes, with a zygosity pattern that matched the expected mode of inheritance. Unsolved cases included patients with negative genetic findings, benign or likely benign variants, variants whose zygosity did not align with the known inheritance pattern (such as a single heterozygous variant in a gene associated with AR disease), or variants detected in genes unrelated to the clinical presentation. Uncertain cases consisted of patients carrying variants of unknown significance (VUS) in IRD-related genes, for whom the available evidence was insufficient to determine pathogenicity.

### 2.4. Investigating Identified Variants

To further investigate cases with variants of uncertain significance, familial segregation analysis was offered to patients and their families. This involved testing family members who agreed to participate to determine whether the variant co-segregated with the disease within the family. Cases that initially posed uncertainty and required family segregation and/or reanalysis of WES data were categorized as “cases reconsidered as likely to be solved”. The initial variant classifications were collected using Franklin, a tool based on the ACMG-AMP guidelines for variant classification (https://franklin.genoox.com/clinical-db/home, accessed on 23 January 2025). The subclassification of VUS variants into categories such as hot, warm, tepid, cool, cold, and ice cold was performed according to the criteria outlined in the Association for Clinical Genomic Science (ACGS) Best Practice Guidelines for Variant Classification in Rare Disease (2020) [16]. We also used Mutation Taster as an additional tool to computationally predict the effect of the variants on the encoded protein (https://www.mutationtaster.org, accessed on 13 May 2024). In addition, Sorting Intolerant From Tolerant (SIFT) and Polymorphism Phenotyping (PolyPhen-2) were used to assess the potential impact of amino acid substitutions on protein function (https://sift.bii.a-star.edu.sg/www/SIFT_dbSNP.html, accessed on 18 May 2024) (http://genetics.bwh.harvard.edu/pph2/index.shtml, accessed on 18 May 2024). The Combined Annotation Dependent Depletion (CADD) score was also utilized to estimate the deleteriousness of each variant. Variants with a CADD score greater than 20 were considered potentially deleterious. PolyPhen-2 scores of 0.85 or higher were classified as “probably damaging,” while SIFT scores of 0.05 or lower were regarded as “deleterious.” Predictions from MutationTaster labeled as “disease causing” were also considered supportive evidence. ClinVar (https://www.ncbi.nlm.nih.gov/clinvar/, accessed on 13 May 2024) was also used to gather data on the clinical significance of the variants and to determine whether a variant had been previously reported in association with IRD-related phenotypes. Population databases such as the Genome Aggregation Database (gnomAD) and the Greater Middle East (GME) (https://gnomad.broadinstitute.org, accessed on 18 May 2024) were utilized to collect data on the allele frequencies of identified variants across different populations.

### 2.5. Investigations on the Variants’ Novelty and PyMol Analysis

We conducted a thorough search of variants in the literature and public databases to identify novel variants. Novel variants were analyzed using PyMOL (Version 3.1.6.1), a software for 3D protein structure visualization. Protein Data Bank (PDB) (https://www.rcsb.org, accessed on 25 July 2024) was utilized for available 3D structures, and if not present, online tools were used to structure the gene variants. The wild-type protein FASTA sequence was initially retrieved from UniProt (https://www.uniprot.org, accessed on 25 July 2024). Following this, the protein sequence was submitted to I-TASSER [17] to predict the crystal structure. Various validation tools were employed to assess the quality of the protein structures. Mutagenesis and alignment analysis were performed on wild-type proteins to evaluate point mutations.

### 2.6. Statistical Analysis

The data collected from all patients were analyzed using the Statistical Package for the Social Sciences (IBM-SPSS v. 28). Frequencies and percentages were computed for categorical variables, such as nationality, gender, consanguinity, and family history. At the same time, the mean and standard deviation were calculated for continuous variables such as age and age of diagnosis. The diagnostic yield was calculated by dividing the number of solved cases by the total number of patients who underwent each genetic test. Cases that were only likely to be solved based on familial segregation, but not definitively confirmed, were excluded from this calculation. Fisher’s Exact test was used to assess the significance of diagnostic yields for each test, with a two-tailed *p*-value of less than 0.05 considered statistically significant.

## 3. Results

### 3.1. Patients’ Demographics and Clinical Characteristics

**Figure 1:**
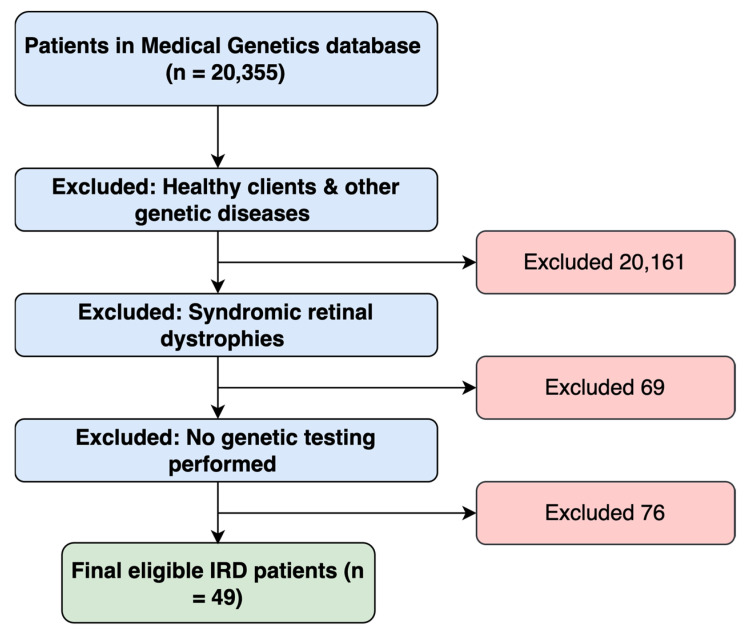
A flowchart demonstrating the approach utilized in identifying eligible study participants.

The database of patients seen at the Department of Adult and Pediatric Medical Genetics at Hamad Medical Corporation included 20,355 patients. Firstly, 20,161 individuals were excluded, as they were patients with other genetic diseases or were healthy clients. In addition, 69 patients with syndromic retinal dystrophy were excluded. Lastly, 76 patients with non-syndromic retinal dystrophy who had not undergone any genetic testing were excluded (Figure 1). Thus, our study identified 49 eligible patients with IRDs and 117 family members from the records of January 2015 to December 2022. Table 1 and Table 2 summarize the participants’ demographic and clinical characteristics.

In our study, 44.9% (*n* = 22) of the participants were males, while 55.1% (*n* = 27) were females (Table 1). The mean age of patients at data collection is 23.35 years, and the mean age at diagnosis is 20.28 years. Of the 49 patients, 90% (*n* = 44) were Arabs, and most of the patients were Qatari, constituting 61.2% (*n* = 30). Patients from other Arab countries were as follows: Egypt 8.2% (*n* = 4), Palestine 8.2% (*n* = 4), Lebanon 4.1% (*n* = 2), Yemen 4.1% (*n* = 2), Syria 2% (*n* = 1), and the United Arab Emirates 2% (*n* = 1). Non-Arab patients included participants from Pakistan (8.2%, *n* = 4) and Croatia (2%, *n* = 1), which together contributed 10.2% (*n* = 5). The consanguinity rate in the study was 79.6% (*n* = 39). Among Qatari participants, the rates were 83.3% (*n* = 25) and 73.6% (*n* = 14) in non-Qatari participants. Of the participants, 67.3% (*n* = 33) had a family history of IRDs. Most patients presented with Rod and Rod-Cone dystrophies, with 21 patients diagnosed with RP, 3 with congenital stationary night blindness, and 1 with Rod-Cone dystrophy. Moreover, 32.7% (*n* = 16) of participants had a clinical diagnosis of Cone and Cone-Rod dystrophies, including five patients with macular dystrophy, four patients with Cone-Rod dystrophy, three patients with Stargardt disease, three patients with Cone dystrophy, and one patient with achromatopsia. Four patients had generalized retinal dystrophy with a diagnosis of LCA. In addition, 8.2% (*n* = 4) of participants had unspecified retinal dystrophy (Table 2).

### 3.2. Genetic Findings

**Figure 2:**
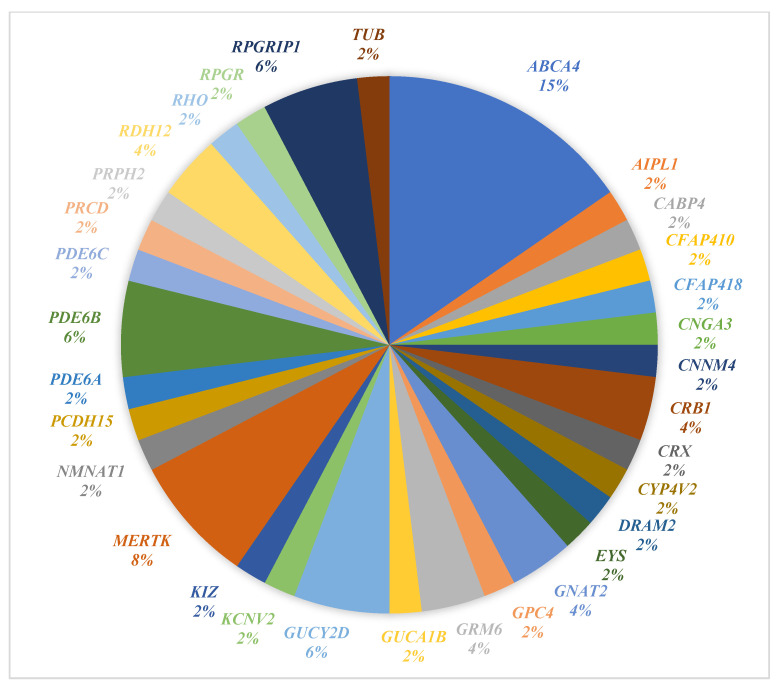
A pie chart illustrating the percentage of identified genes (with genetic variants) among our entire patient cohort (*n* = 49).

**Figure 3:**
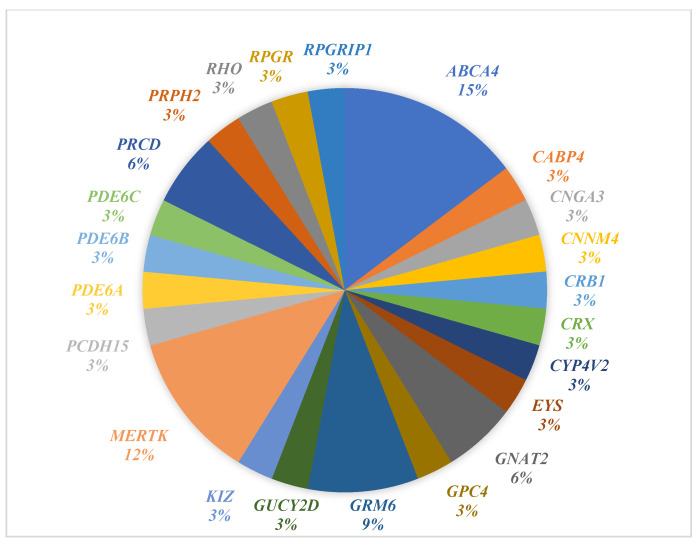
A pie chart demonstrating the percentage of identified genes (with genetic variants) specifically among Qatari nationals in our cohort (*n* = 30).

In our study, we identified 55 variants across 32 genes associated with IRD (Figure 2). Figure 3 illustrates the distribution of identified genes harboring variants among the Qatari participants in our cohort (*n* = 30). Identified variants were found in 46 out of the 49 patients examined. The most reported genes were *ABCA4*, *CRB1*, *GNAT2*, *GRM6*, *GUCY2D*, *MERTK*, *PDE6B*, *RDH12*, and *RPGRIP1*. The *ABCA4* gene was the most reported in the study, with eight patients carrying seven different *ABCA4* variants. According to genetic test reports, out of the 55 identified variants, 48 were inherited in an AR manner, 4 in an AD manner, 2 in an XL manner, and one had an unknown inheritance pattern.

### 3.3. Solved Cases

In the current study, 34 out of 49 cases were initially classified as “solved,” as the genetic test identified a pathogenic or likely pathogenic causative variant in genes related to IRD, thereby explaining the patient’s phenotype. A total of 22 genes and 35 variants were identified in the solved group (Table 3). The most frequently reported gene in the solved cases was *ABCA4*, which was identified in six patients. To a lesser extent, the genes *MERTK*, *PDE6B*, *GRM6*, *RDH12*, *RPGRIP1*, and *GNAT2* were all reported in at least two patients from our cohort. All patients were found to have causative variants in a single gene except for three patients—IRD-1, IRD-9, and IRD-47—who were found to have causative variants in two genes. Patient IRD-1 was homozygous for the pathogenic variant c.2214delT in the *MERTK* gene and for the pathogenic variant c.1040_1041delTT in the *GUCY2D* gene. IRD-9 was compound heterozygous for the pathogenic variant c.5882G>A and the likely pathogenic variant c.1609C>T in the *ABCA4* gene, and heterozygous for the pathogenic variant c.128G>A in the *CRX* gene. IRD-47 was heterozygous for the pathogenic variant c.5882G>A in the *ABCA4* gene and homozygous for the pathogenic variant c.1348C>T in the *CYP4V2* gene. Thirty-two variants were inherited in an AR pattern, two variants as AD, and one variant as XL. Of the 34 solved cases, 3 were compound heterozygous. Familial segregation studies were performed for the cases with compound heterozygous variants. In these cases, each parent was identified as a carrier for one of the variants. However, there was an exception in the case of patient IRD-35 from Lebanon. The patient was compound heterozygous for the likely pathogenic c.3278dupC variant and the pathogenic variant c.2935C>T in *RPGRIP1*. Parental testing revealed that one variant (c.2935C>T) was inherited from the mother. At the same time, the other was not detected in either parent and is likely to have occurred spontaneously (either de novo or due to germline mosaicism). Family segregation studies also showed that in patient IRD-9 with three identified variants, the pathogenic variant c.5882G>C in the *ABCA4* gene was inherited from the father. In contrast, the likely pathogenic variant c.1609C>T in the *ABCA4* gene was inherited from the mother. However, the pathogenic variant c.128G>A in the *CRX* gene was not detected in either parent, indicating that the variant is possibly de novo. RP was the most common clinical manifestation (*n* = 17), while Achromatopsia was the least common, reported in only one patient. Additionally, 12 cases were initially classified as uncertain based on the ACMG criteria, with their variants categorized as VUS. Following the family segregation analyses, 5 cases were reconsidered as likely to be solved as the variants segregated with the disease in family members.

### 3.4. Unsolved Cases

Unsolved cases included three participants, two of whom had negative genetic test results (1 had WES Plus conducted, and 1 had gene panel testing). One patient, IRD-26 from Palestine, underwent gene panel testing and was found to be heterozygous for two AR variants, c.8177_8187del in *ALMS1* and c.190+2T>C in *MKS1*, both of which are related to syndromic retinal dystrophy. These findings, however, did not explain the patient’s phenotype.

### 3.5. Uncertain Cases

Twelve participants were initially categorized as uncertain cases, with 18 identified variants in 13 genes (Supplemental Tables S2 and S3). The most frequently reported gene was *ABCA4* (2 patients). Among the 12 patients, two participants had variants identified in two different genes. Patient IRD-34 was heterozygous for the risk allele c.5603A>T in *ABCA4* and homozygous for a variant of uncertain significance (VUS), c.246T>G in *DRAM2*. Patient IRD-17 was homozygous for a VUS, c.103G>A in *PDE6A*, and hemizygous for the variant c.156C>G in *GPC4* (Supplemental Table S3). In this group, 15 variants were inherited in an AR manner, two as AD, and one as XL. Family segregation analyses were conducted (Supplemental Figure S1), testing other family members of the probands for the same identified variant, which may help assess the clinical significance of uncertain test results. Following family segregation studies, five cases were reconsidered as likely to be solved, as the genetic variant segregated with the disease among family members (Supplemental Table S2). Out of the 12 cases, five were reconsidered as likely to be solved, while seven remained uncertain (Supplemental Table S3).

### 3.6. Shared vs. Novel Variants

In total, 49 of the identified variants had been previously reported, while six were novel (Supplemental Table S4). Out of the 55 variants, 13 had previously been reported in other patients, as documented in the literature, population databases, and public variant archives. Some variants were identified in individuals with similar ethnic backgrounds to those of our study participants. For example, the c.2214delT variant in the *MERTK* gene was identified in two Qatari patients. It had also been reported in patients from Saudi Arabia and the United Arab Emirates, all of whom exhibited similar clinical manifestations [3,20]. Additionally, patient IRD-4 from Qatar shared the variant c.81_82insA in the *CABP4* gene with patients from Saudi Arabia [22]. Another shared variant, c.821T>C in the *RDH12* gene, was identified in two of our patients from Palestine and has been reported in patients with similar ethnic backgrounds [17] (Supplemental Table S4). Conversely, several variants were identified in patients from diverse ethnic backgrounds. For example, the c.2137+1G>A variant in the *EYS* gene was reported in a Qatari patient in our study and a patient from Denmark [18]. The variant c.5882G>A in the *ABCA4* gene was reported in three Qatari patients in our study and has also been observed in patients from multiple ethnicities, including those from China, Spain, the United Arab Emirates, and Italy [3,18,19,21]. The variant with the highest frequency was c.5603A>T in the *ABCA4* gene, with a heterozygous/homozygous frequency of 0.04042 in GnomAD and 0.039314516 in GME, and was associated with a complex retinal dystrophy phenotype on ClinVar (Supplemental Table S4). We identified five patients with six novel variants in 4 genes that had not been previously reported (Supplemental Table S5). The two most frequently reported genes were *RPGRIP1* (c.3278dupC and c.105dupA) and *CRB1* (c.3613G>T and c.4211G>C), each containing two novel variants (Supplemental Table S5). All novel variants were inherited in an AR pattern. Of the five patients, three were homozygous, and 2 were compound heterozygous. IRD-36 was found to be compound heterozygous for two novel variants in the *CRB1* (c.3613G>T and c.4211G>C). At the same time, IRD-35 was compound heterozygous for one novel variant, c.3278dupC, and one shared variant, c.2935C>T, in the *RPGRIP1* gene. Prediction databases were utilized to assess the potential effects of these variants on protein structure and function. All six novel variants were found to be disease-causing (Supplemental Table S5).

### 3.7. Molecular Visualization Analysis

Molecular visualization analysis of the novel variants was performed using PyMol (Supplemental Table S5). Predicted models of wild-type proteins were selected based on C-score, a measure of protein quality derived from alignment analysis. Accepted C-score levels for protein models typically range between -5 and 2, with higher values indicating better model quality. These models were further evaluated using various validation tools (Supplemental Table S6). Novel variants identified were possibly causing steric hindrance and consequent destabilization of the protein structure. Supplemental Figures S2–S7 illustrate the main findings of the molecular visualization analysis, including the wild-type protein structure, the affected area of the protein structure, and the mutant protein structure. As shown in Supplemental Figure S2, the loss of polar contacts is predicted to occur in p.Arg1404Thr in the *CRB1* gene, which could destabilize the protein structure. The alignment analysis between the *CRB1* wild-type protein and the mutant Arg1404Thr *CRB1* protein structure, which measures the structural congruence between the two, demonstrates an exceptionally close correspondence, as evidenced by a Root Mean Square Deviation (RMSD) value of 0.0. RMSD, a metric quantifying the average spatial deviation between equivalent atoms in two protein structures, is a vital tool for assessing structural variations and, in this context, confirms the near-identical nature of the wild-type and mutant protein structures On the other hand, the alignment between the wild-type *GUCY2D* and its mutant p.Glu738del protein structure showed slight misalignment in the overall protein structure with an RMSD value of 1.957 (Supplemental Figure S7).

### 3.8. Test Frequency and Diagnostic Yield

To assess the diagnostic yields of different genetic tests, Fisher’s exact test was used to determine if a significant association existed between the type of genetic test and the number of solved cases, as this test best represents the diagnostic yield (Supplemental Table S7). All participants in the study underwent a single genetic test, with the most common being WES Plus. Of the 49 patients, 14 had WES, 8 had gene panel testing, 4 underwent familial targeted testing, and 23 received WES Plus. Twenty-five cases were solved by WES and WES Plus, six by gene panel testing, and three by familial targeted testing. No significant association was detected through statistical analysis (Supplemental Table S7).

## 4. Discussion

Our study investigated the genetic factors underlying non-syndromic IRD at the Department of Adult and Pediatric Medical Genetics, Hamad Medical Corporation, between 2015 and 2022. We identified 49 eligible patients with 55 variants across 32 different IRD-related genes.

Qatari patients comprised the majority (61.2%, *n* = 30), while other Arab patients made up 28.4% (*n* = 14). The consanguinity rate in our cohort was approximately 78%, similar to the 68% reported in previous IRD studies on Saudi Arabian patients [20]. Among the 55 identified variants, 48 followed an AR inheritance pattern. This finding is consistent with the high consanguinity rate observed in our patient population (79.6%), which significantly exceeds the national average of 56% [23]. Among the 49 participants, 67.3% (*n* = 33) had a positive family history of IRD, compared to 35% reported in cases from England. This discrepancy is likely attributable to the higher rates of inbreeding and consanguinity observed in Qatar [24]. Retinitis pigmentosa was the most common clinical diagnosis among our participants, a pattern similarly observed in other Arab countries, such as Saudi Arabia (55%) [25], as well as in European countries like Denmark (24%) [18].

### 4.1. Genetic Testing Options

A total of 76 clinically diagnosed non-syndromic IRD patients were excluded from the analysis due to the absence of genetic testing results. While this exclusion was necessary to ensure the accuracy and interpretability of genetic data, it may have introduced a selection bias, as patients with access to advanced genetic testing are often those with stronger healthcare accessibility or referral opportunities. All participants in our study underwent one genetic test, with the most commonly used test being WES Plus. Out of the 49 patients, 14 underwent WES, 8 underwent gene panel testing, 4 underwent familial targeted testing, and 23 underwent WES Plus. We found that 70% (*n* = 35) of patients with unknown familial pathogenic variants opted for WES as their preferred genetic testing method, as opposed to gene panel testing. This preference is due to the comprehensive nature of WES and the common practice at HMC of including genetic data from other family members when conducting WES. This approach enables a more thorough analysis, facilitating the identification of shared genetic variants among family members and thus allowing for the diagnosis of multiple family members with a single test.

Additionally, WES testing is typically covered by the government for Qatari patients, making it their preferred initial testing method over a stepwise approach. Patients who underwent gene panel testing in earlier years had limited and less comprehensive panels. However, those tested more recently had access to more comprehensive gene panels, which included some that incorporated parental samples. Mitochondrial genome testing in IRD can be used to capture cases where mitochondrial genetic variants may contribute to the patient’s phenotype. However, in our study, mitochondrial genome testing did not identify any causative variants. This finding aligns with previous studies, which report that mitochondrial variants are relatively rare compared to other genetic causes of IRD [26]. We did not find a significant association between the type of genetic test and the diagnostic yield. Gene panel testing demonstrated an initial diagnostic yield of 75%, while WES yielded a comparable rate of 67.6%, before incorporating the results of family segregation analyses. A previous genetic investigation of hereditary retinal disorders in Sweden, using a 322-gene panel, reported a molecular diagnosis in 65% of patients [27]. Another study in Saudi Arabia reported higher diagnostic yield rates (up to 82%) for referred patients using WES [20]. Variations in the genes included in different gene panels could explain the observed differences in diagnostic yields. In our study, the higher diagnostic yield observed with WES compared to gene panel testing may be attributed to the broader nature of WES, which increases the likelihood of detecting pathogenic or novel variants across a broader range of genes, including those not yet included in standard gene panels. It is worth noting that the number of uncertain cases was higher with WES, which is expected given its broader scope compared to targeted gene panel testing.

### 4.2. Genetic Test Results

Among the 32 identified genes, the *ABCA4* gene was the most frequently reported. Similar findings were observed in studies from the United Arab Emirates and the United Kingdom, where *ABCA4* was also the most reported gene [3,28]. Other commonly reported genes in our study included *CRB1*, *GNAT2*, *GRM6*, *GUCY2D*, *MERTK*, *PDE6B*, *RDH12*, *and RPGRIP1*, each of which was detected in at least two patients. Of the 55 identified variants, 48 were inherited in an AR manner, four were AD, two were X-linked, and 1 had an unknown inheritance pattern. Of the 55 observed variants, the majority (*n* = 36; 64.55%) were homozygous for the variant identified. This is consistent with the fact that 79.6% of the patients came from consanguineous families [23].

### 4.3. Solved Cases

Thirty-four cases were initially classified as solved, as genetic testing identified a pathogenic or likely pathogenic variant in IRD-related genes. *ABCA4* was the most frequently reported gene in this group, identified in six patients. This can be attributed to the critical role of the *ABCA4* protein in proper retinal function [29]. Pathogenic variants in the *ABCA4* gene are a significant cause of AR cone-rod dystrophy, accounting for 30 to 60% of cases, including Stargardt disease. Patients with these variants may exhibit clinical manifestations such as Stargardt disease and RP. Stargardt disease is the primary clinical manifestation associated with *ABCA4* variants. The clinical presentation of *ABCA4* variants is heterogeneous, with retinal degeneration varying in severity and primarily affecting the macula or peripheral retina. This variability is indicative of a spectrum of a unified disease process known as *ABCA4*-related retinopathy [30]. Previous studies on IRD patients from Saudi Arabia and the United Arab Emirates also identified *ABCA4* as a major contributor to IRD in their patients [3,20].

In contrast, *RPGR* is the most common cause of IRD in the Chinese population [31]. The second most frequently reported gene in our study was *MERTK*, detected in 4 Qatari patients with RP. The prevalence of *MERTK* variants in IRD patients varies across different populations [32]. In contrast to our findings, *MERTK* variants are generally considered rare causes of IRD, accounting for only around 1% of cases [33]. Several studies have reported the prevalence of *MERTK* variants in different populations. For example, a study of Japanese patients with RP found that *MERTK* variants accounted for 3.6% of cases [34]. A study of North African patients supports our findings on the role of *MERTK* variants in IRD. According to Jaffal et al. (2021), *MERTK* variants accounted for 18% of IRD cases studied [13].

### 4.4. Unsolved Cases

Two patients (IRD-5 and IRD-27) had negative genetic test results, and one patient (IRD-26) was heterozygous for two variants in distinct AR genes, *ALMS1* and *MKS1*. Patient IRD-5, diagnosed clinically with Leber congenital amaurosis (LCA), underwent WES Plus testing, which yielded negative results. This may reflect one of the known technical limitations of whole-exome sequencing, which does not effectively detect deep intronic, regulatory, or structural variants located outside the protein-coding regions [35]. It is therefore possible that this patient carries a pathogenic variant in a non-coding region, which would require whole-genome sequencing (WGS) or long-read sequencing for confirmation. Patient IRD-27, a 60-year-old Qatari female clinically suspected of adult-onset vitelliform macular dystrophy (AVMD), also had negative results following targeted gene panel testing. While AVMD was initially described as a dominantly inherited disease primarily associated with pathogenic variants in *PRPH2*, *BEST1*, *IMPG1*, *and IMPG2* [36], recent reports have demonstrated that a substantial proportion of AVMD cases remain genetically unexplained. Many of these are now considered idiopathic or multifactorial, suggesting the involvement of as-yet unidentified genes or environmental modifiers [36]. This could explain the lack of a molecular diagnosis in IRD-27 despite a strong clinical phenotype. Patient IRD-26 presented with variants in two genes, *ALMS1* and *MKS1*, which are typically linked to syndromic ciliopathies, including Alström syndrome, Meckel syndrome, Bardet–Biedl syndrome, and Joubert syndrome. However, both variants were found in the heterozygous state and therefore insufficient to establish a diagnosis consistent with the clinical presentation. Notably, previous studies have reported that heterozygous pathogenic variants in syndromic IRD-associated genes can occasionally manifest as isolated non-syndromic retinal disease, likely due to variable penetrance, modifier genes, or compound heterozygosity with undetected variants [22]. These observations underscore the complexity of genotype–phenotype correlations in IRDs and highlight the importance of combining clinical reassessment with advanced sequencing approaches in unresolved cases.

### 4.5. Uncertain Cases

Out of 12 uncertain cases, five were reclassified as solved through family segregation. In family segregation studies, it was found that the identified variants segregated with the disease within the family. For instance, patient IRD-7 was homozygous for the *PDE6B* gene variant c.2407A>G, and family segregation showed both parents and healthy siblings were heterozygous carriers.

Another patient, IRD-48, underwent familial targeted testing for the *CNNM4* gene variant c.509T>C, which had been previously identified in two relatives with IRD. Despite this, the variant remained classified as of uncertain significance, underscoring the need for functional studies to more accurately predict its impact.

These findings underscore the importance of conducting family segregation analysis when family members are available to assist in resolving uncertain cases in IRD genetic diagnosis. As clinical genetic testing evolves from diagnostic to predictive, documenting such variants can aid in disease management, offering options like pre-implantation genetic testing (PGT). Genetic counselors play a crucial role in interpreting variant data and familial segregation evidence, providing patients with valuable information for family planning and handling the emotional and psychological aspects of the test results [35,37].

Out of the 12 cases, seven remained uncertain with identified variants of uncertain significance. Previous studies indicate that uncertain genetic variants can add complexity to clinical decision-making and result in harm and costs to patients and the healthcare system [38]. While efforts to improve variant interpretation are ongoing, VUSs continue to pose a challenge due to the high prevalence of rare and novel variants in the human genome. Strategies to mitigate these challenges include limiting the identification of VUSs, subclassifying them based on harm likelihood, family-based evaluations, and enhanced counseling [38].

### 4.6. Molecular Visualization of the Six Novel Variants

The predicted structural effects of the six novel variants demonstrate biologically meaningful patterns that align closely with both the severity of the clinical phenotype and the zygosity of the affected individuals. The *RPGRIP1* frameshift variant p.Pro36Thrfs35 produced the most substantial disruption, with only 36 amino acids translated from a protein that normally contains 1286 residues. This extreme truncation eliminates nearly all functional domains required for the RPGRIP1–RPGR interaction at the photoreceptor connecting cilium, providing a strong biological explanation for the patient’s severe LCA phenotype in the homozygous state. In contrast, the *RPGRIP1* p.Gln1094Thrfs6 variant produced a much smaller truncation limited to the C-terminal region of the protein, preserving the RPGR-interacting domain. This milder structural impact is consistent with the less severe rod–cone dystrophy observed in the patient, demonstrating a clear correlation between the extent of predicted structural disruption and clinical severity.

The *GUCY2D* p.Glu738del variant exhibited an RMSD of 1.957 when aligned with the wild-type protein, indicating only a slight structural deviation. Although structural modeling suggested minimal conformational distortion, the homozygous state of this variant and the established sensitivity of *GUCY2D* to even subtle alterations in the catalytic or regulatory domains support its likely pathogenic role in LCA. This illustrates that, for some genes with well-defined functional constraints, modest structural alterations can still translate into clinically significant phenotypes when combined with zygosity and known gene–disease mechanisms. For *CRB1*, the missense variant p.Arg1404Thr showed no measurable change in overall protein structure (RMSD = 0), and was therefore classified as a VUS. However, the patient presented with cone-rod dystrophy, which is biologically explained by the presence of this variant in a compound heterozygous state alongside a second, clearly pathogenic truncating variant (p.Gly1205Ter). The combined effect of a truncating allele with a potentially mild missense substitution is consistent with the gene’s known dosage sensitivity and the requirement for two dysfunctional alleles to manifest disease, highlighting how compound heterozygosity can produce a phenotype even when one allele appears structurally benign. Taken together, these findings emphasize that the biological relevance of predicted structural changes becomes evident when integrated with gene function, known protein domains, zygosity, and clinical expressivity. Structural modeling alone may underestimate the functional consequences of some variants, but in combination with genetic and clinical evidence, it provides a coherent and biologically robust explanation for the observed disease phenotypes. It is essential to acknowledge that inherited alterations in protein structure can cause diverse effects on protein morphology and function [39]. Additionally, variants within the same gene may exert distinct structural consequences, leading to a spectrum of retinal diseases [39]. To validate these findings comprehensively, in vitro studies and animal models are required.

### 4.7. Genotype-Phenotype Correlation

In the current study, the *ABCA4* gene was the most frequently reported gene, with considerable variability observed in the associated clinical manifestations and age of onset among affected individuals. Patient IRD-9, a 14-year-old with a clinical diagnosis of Stargardt disease, was found to be compound heterozygous for c.1609C>T and c.5882G>A in the *ABCA4* gene. In comparison, patients IRD-22, a 56-year-old, and IRD-47, a 69-year-old, with a clinical diagnosis of RP, were both found to be heterozygous for the variant c.5882G>A in the *ABCA4 gene*. This variability in disease severity and age of onset can be explained by previous studies, which have reported that biallelic pathogenic variants in the *ABCA4* gene are associated with Stargardt disease, typically presenting in the first or second decade of life [40]. At the same time, heterozygous pathogenic variants in *ABCA4* have also been reported in association with age-related macular degeneration type 2 (ARMD2), which typically manifests later in life (43–45 years).

Patients IRD-1 and IRD-21 were both homozygous for the c.2214delT variant in the *MERTK* gene and had a clinical diagnosis of RP. This variant was also observed in RP patients from Saudi Arabia and the United Arab Emirates [3,41]. Participant IRD-8 was homozygous for the c.5584G>C variant in the *ABCA4* gene and exhibited a Stargardt disease phenotype, a condition also reported in patients from Sweden [27]. These findings suggest that specific variants exhibit similar behavior and result in comparable clinical phenotypes. However, a few participants had clinical diagnoses that differed from those reported in the literature. Patient IRD-48 was clinically diagnosed with cone-rod dystrophy and was found to be homozygous for the variant c.509T>C in the *CNNM4* gene. A similar phenotype was reported in patients from Saudi Arabia [41]. However, the same variant was seen in 1 patient from the United Arab Emirates with a clinical diagnosis of Jalili syndrome [3], indicating pleiotropic effects of the variant. The variant c.81_82insA in *CABP4* was identified in a Qatari participant from our study, but the ophthalmologists did not specify his clinical diagnosis. This variant was reported in 11 patients from Saudi Arabia, all of whom exhibited similar ophthalmic phenotypes, which were classified as cone–rod synaptic disorder [21].

Some identified variants had a possible founder effect. The *MERTK* gene variant c.2214delT was identified in two individuals from Qatar and has also been reported in other studies on patients from Saudi Arabia and the United Arab Emirates, who exhibited similar clinical symptoms [3,26]. Two patients from Palestine also had the *RDH12* gene variation c.821T>C, which has been described in the literature as a founder variant in Arabs and Bedouins and was observed in individuals from Palestine [17]. Founder effect variants are well-known phenomena in different populations. Endogamy and consanguinity are cultural practices that increase the likelihood of homozygosity for genetic variants, thereby contributing to the prevalence of recessive conditions [42]. These genes are associated with specific clinical manifestations and should therefore be considered as first-tier testing for individuals residing in regions with these characteristics. The *ABCA4* gene variant c.5882G>A is one of the most reported IRD variants worldwide and has been identified across diverse populations. [43]. This variant was also identified in 3 Qatari patients from our cohort and was linked to the identifiable spectrum of Stargardt disease. Given its widespread prevalence, it is recommended that individuals with similar clinical characteristics should be tested for this variant, regardless of geographic region.

Moreover, difficulties in identifying the most appropriate genetic test may stem from the fact that referrals in this cohort came from various ophthalmologists, each with distinct approaches to identifying and classifying inherited retinal diseases. Previous studies have reported a higher diagnostic yield when a single ophthalmologist with expertise examined and confirmed the diagnoses in each patient, and when a clinical diagnosis as specific as possible was obtained, thereby increasing the diagnostic yield [3]. This highlights the importance of genotype–phenotype correlation in selecting the most suitable genetic test for a given individual.

### 4.8. Therapeutic Options

The spectrum of variants identified in our cohort offers valuable insights into molecular diagnostics and potential gene therapy in patients with IRD. None of the study participants had the *RPE65* gene variant. Novel therapies for inherited IRDs have rapidly emerged, particularly since clinical trials for LCA caused by *RPE65* variants led to the first FDA-approved in vivo gene therapy [44]. Stargardt disease, caused by pathogenic variants in the *ABCA4* gene, was the most frequently reported gene in our cohort [45]. Stargardt disease is a common cause of childhood blindness, though gene therapy has not yet achieved clinical trial success comparable to that of other hereditary retinal diseases [45]. However, Stargardt disease appears responsive to therapeutic intervention due to its early age of onset and ongoing disease progression throughout an individual’s lifetime [45]. Previous clinical trials for Stargardt disease gene therapy have demonstrated that EIAV-ABCA4 subretinal therapy is well-tolerated, with only one instance of ocular hypertension. Macular flecks were also significantly reduced in the treated eye. To fully describe the safety and effectiveness of EIAV-ABCA4, more patient testing and follow-up will be necessary [45]. These findings underscore the importance of investigating the prevalence of ABCA4 gene variants in Qatar, as gene therapy may become a viable option in the future.

Moreover, advances in viral vectors have led to more effective Adeno-associated virus (AAV) transduction and the development of new viral vectors for the gene augmentation treatment of large gene targets. Rod-cone dystrophies (RCD), the most reported phenotype in our cohort, are characterized by the progressive loss of rod photoreceptors, followed by cone photoreceptor degeneration, ultimately leading to blindness [46]. RCD affects more than 1.5 million people worldwide, and over 65 genes are involved [47]. The *NXNL1* gene encodes proteins generated by the photoreceptors. For instance, a new AAV-based therapeutic candidate called SPVN06 encodes human retinal proteins in the same vector. A single subretinal injection of SPVN06 is expected to prevent cone degeneration in RCD patients, regardless of the specific mutated gene [47]. Such findings can present new therapeutic options for patients with IRD in Qatar.

### 4.9. Qatar’s IRD Genetic Landscape in the Context of Arab Populations

Our findings in Qatar largely reflect patterns reported in other Arab countries. Similarly to our cohort, studies from Saudi Arabia and the UAE consistently identify *ABCA4* as a major IRD gene, with variants such as c.5882G>A strongly linked to Stargardt disease and cone-rod dystrophy [48]. *ABCA4* is a gene well known for its numerous founder alleles, each with characteristic geographic distributions. Its mutational spectrum varies substantially across ethnic backgrounds, and certain variants demonstrate clear regional enrichment. For example, the globally common *ABCA4* variant c.5882G>A (p.Gly1961Glu), which is believed to have originated in Eastern Africa and subsequently dispersed through population migration, is also the most frequent *ABCA4* variant identified in our cohort. Similar patterns have been reported across multiple Arab populations, where distinct founder alleles or locally enriched variants reflect shared ancestry, historical migration, and long-standing consanguinity [48]. Therefore, the high prevalence of *ABCA4* variants observed in our study—especially c.5882G>A—likely reflects a combination of regional founder effects and shared genetic background with neighboring Middle Eastern and North African populations [48]. Other genes detected in our cohort, including *RPGRIP1*, *CNNM4*, and *CABP4*, have similarly been reported in Saudi and UAE populations, often showing pleiotropic effects that complicate diagnosis [48].

These genetic overlaps reflect shared cultural and social practices, particularly high consanguinity and endogamy, which remain prevalent across Arab and Muslim societies due to strong family structures and religious traditions [49]. Such practices increase homozygosity for AR variants in the region. Consequently, genetic testing and counseling are critical for early diagnosis and informed reproductive decisions. Recent studies indicate growing awareness and positive attitudes toward genetic counseling among Arab families. However, misconceptions and stigma persist, often influenced by cultural norms and concerns about privacy and marriage prospects [50].

Regionally, several initiatives aim to address these challenges. The Qatar Genome Program (QGP), launched under the Qatar Precision Health Institute, has sequenced thousands of Qatari genomes to identify population-specific variants, including those linked to IRDs, and integrates these findings into clinical care and preventive strategies [50]. Similarly, the Emirati Genome Program analyzed over 500,000 genomes to improve variant interpretation and penetrance estimates for IRDs [51], while Saudi Arabia continues to expand clinical genomics services and public awareness campaigns [52]. These efforts underscore the importance of integrating genomics into public health and promoting culturally sensitive counseling strategies to enhance acceptance and uptake across Arab populations.

### 4.10. Limitations and Future Directions

Our study had several limitations, including the retrospective design and the relatively small sample size, as many patients did not undergo genetic testing due to personal or financial reasons, which limited our statistical power. Additionally, several participants lacked a precise clinical diagnosis of IRD from ophthalmologists. This underscores a broader limitation of the study, as it lacked comprehensive clinical data across all participants, making it difficult to assess the full extent of genotype-phenotype variability. Future research should enhance the collection of detailed clinical data and investigate potential modifying factors that may contribute to these phenotypic differences, including environmental influences and epigenetic factors. Although family segregation studies were conducted and provided valuable insights, they may not always be sufficient to definitively classify certain cases as solved, particularly in the absence of functional validation. This limitation highlights the need for caution when interpreting results based solely on segregation studies without additional supporting evidence. Future directions in this field should focus on expanding the sample size to increase statistical power and conducting more comprehensive genetic testing to identify potential causative variants in patients who did not undergo testing in this study. Investigating why some patients declined genetic testing could help improve patient education and counseling. Additionally, efforts should be made to ensure precise and accurate clinical diagnoses, thereby improving the ability to establish genotype-phenotype correlations. It may also be beneficial to investigate potential environmental factors that contribute to IRD.

## 5. Conclusions

In conclusion, we identified 55 variants across 32 IRD-related genes in 49 patients. Of the 49 cases, 32 were solved. Rod-dominated phenotypes account for a significant percentage (51%) of hereditary retinal diseases in our study cohort. Specific genes have recurrent variations that are most likely the result of regional founder effects, such as the variant c.821T>C in the *RDH12* gene identified in Palestinian patients and the variant c.2214delT *in the MERTK* gene identified in the Arabian Peninsula’s population. The study also highlighted that both WES and gene panel testing showed similar diagnostic yields within our research. There is a clear preference for WES as the first-tier test due to its reduced turnaround time. Moreover, family segregation studies play a significant role in identifying possible causative variants. Our study expands the understanding of the genetic heterogeneity of IRD among the Arabian population. This work expands our understanding of the molecular mechanisms underlying IRD, while also facilitating the development of personalized treatment options and providing accurate genetic counseling services for patients and their families. More investigation in the region is required before generalizations. This is the first study of its kind conducted in Qatar, laying the groundwork for further research on the epidemiology and genetics of IRD in the region.

## Figures and Tables

**Table 1:** Demographic Data of the Study Cohort (*n* = 49).

Variable/Diagnosis	Count/Mean	Percentage/SD
**Age**		
Age at Data Collection (years)	23.35	±18.73
Age at Genetic Diagnosis (years)	20.28	±18.69
**Gender**		
Female	27	55.1%
Male	22	44.9%
**Nationality**		
**Total Arabs**	44	89.7%
Qatar	30	61.2%
United Arab Emirates	1	2.0%
Yemen	2	4.1%
Lebanon	2	4.1%
Palestine	4	8.2%
Syria	1	2.0%
Egypt	4	8.2%
**Total non-Arab**	5	10.2%
Pakistan	4	8.2%
Croatia	1	2.0%
**Consanguinity**		
Yes	39	79.6%
No	6	12.2%
Not Available	4	8.2%
**Family History**		
Positive	33	67.3%
Negative	13	26.5%
Not Available	3	6.1%

**Table 2:** Clinical Diagnosis of the Study Cohort (*n* = 49).

Diagnosis	Count	Percentage
**Cone-related Disorders**	**8**	**16.3%**
Cone dystrophy	3	6.1%
Cone-Rod dystrophy	4	8.2%
Achromatopsia	1	2.0%
**Macular Disorders**	8	**16.3%**
Stargardt disease	3	6.1%
Macular dystrophy	5	10.2%
**Rod-related Disorders**	**22**	**44.9**
Retinitis pigmentosa	21	42.9%
Rod-Cone dystrophy (non-RP)	1	2.0%
**Early-Onset/Severe Congenital Disorders**	**7**	**14.3%**
Congenital stationary night blindness	3	6.1%
Leber congenital amaurosis	4	8.2%
**Unspecified retinal dystrophy**	4	8.2%

**Table 3:** Identified causative variants in the solved group.

Patient ID	Country of Origin	Age (Years) *	Age of Diagnosis (Years)	Patient Phenotype	*Gene*	Gene (NM Number)	rsID	Variant (cDNA)	Variant (Protein)	Variant Type	Variant Impact	Zygosity	Pattern of Inheritance	Test Performed	ACMG	ACMG Highest Pathogenicity Evidence	VUS Subclassification	Mutation Taster	Reports from Other Populations/Ethnicities	Reported Phenotype	References
**IRD-1**	Qatar	2	3 months	RP	*GUCY2D*	NM_000180.4	rs763890649	c.1040_1041delTT	p.Phe347TrpfsX5	Deletion	Frameshift	Homozygous	AR	WES	Pathogenic	PVS1	_	Disease causing			
					*MERTK*	NM_006343.3	rs886039422	c.2214delT	p.Cys738TrpfsX32	Deletion	Frameshift	Homozygous	AR		Pathogenic	PVS1	_	Disease causing	Saudi Arabia, UAE	RP & Rod cone dystrophy	[3,17]
**IRD-21**	Qatar	34	28	RP	*MERTK*	NM_006343.3	rs886039422	c.2214delT	p.Cys738TrpfsX32	Deletion	Frameshift	Homozygous	AR	Familial targeted testing	Pathogenic	PVS1	_	Disease causing	Saudi Arabia, UAE	RP & Rod cone dystrophy	[3,17]
**IRD-2**	Qatar	3	3	Congenital stationary night blindness	*GRM6*	NM_000843.4	rs752205220	c.1478G>A	p.Trp493Ter	Substitution	Nonsense	Homozygous	AR	WES	Pathogenic	PVS1	_	_	_		_
**IRD-6**	Qatar	6	6	LCA	*GRM6*	NM_000843.4	rs752205220	c.1478G>A	p.Trp493Ter	Substitution	Nonsense	Homozygous	AR	WES Plus	Pathogenic	PVS1	_	_	_		_
**IRD-4**	Qatar	4	6 months	Uncategorized Retinal Dystrophy	*CABP4*	NM_145200.5	rs786205852	c.81_82insA	p.Pro28ThrfsX4	Substitution	Frameshift	Homozygous	AR	WES Plus	Pathogenic	PVS1	_	Disease causing	Saudi Arabia	Segregated with congenital retinal dysfunction in 11 affected individuals (aged 2–26 years) from four consanguineous families	[18]
**IRD-8**	Qatar	26	23	Stargardt Disease	*ABCA4*	NM_000350.3	_	c.5584G>C	p.Gly1862Arg	Substitution	Missense	Homozygous	AR	WES Plus	Pathogenic	PS4	_	Disease causing	China	Stargardt disease	[19]
**IRD-9**	Qatar	14	11	Macular Dystrophy	*ABCA4*	NM_000350.3	rs61748556	c.1609C>T	p.Arg537Cys	Substitution	Missense	Heterozygous	AR	WES Plus	Likely Pathogenic	PM1	_	_	Germany	Stargardt disease	[20]
							rs1800553	c.5882G>A	p.Gly1961Glu	Substitution	Missense	Heterozygous	AR		Pathogenic	PS3	_	_	China, Spain, UAE, Italy	Stargardt disease	[3,19,21]
					*CRX*	NM_000554.6	rs771736389	c.128G>A	p.Arg43His	Substitution	Missense	Heterozygous	AD		Pathogenic	PS4	_	Disease causing			
**IRD-25**	Yemen	20	18	Stargardt Disease	*ABCA4*	NM_000350.3	rs61750155	c.4793C>A	p.Ala1598Asp	Substitution	Missense	Homozygous	AR	WES Plus	Pathogenic	PM3	_	_	Germany	Stargardt disease	[22]
**IRD-22**	Qatar	56	53	RP	*ABCA4*	NM_000350.3	rs1800553	c.5882G>A	p.Gly1961Glu	Substitution	Missense	Heterozygous	AR	WES	Pathogenic	PS3	_	_	China, Spain, UAE, Italy	Stargardt disease	[3,19,21,23]
**IRD-47**	Qatar	69	64	RP	*ABCA4*	NM_000350.3	rs1800553	c.5882G>A	p.Gly1961Glu	Substitution	Missense	Heterozygous	AR	WES Plus	Pathogenic	PS3	_	_	China, Spain, UAE, Italy	Stargardt disease	[3,19,21,23]
					*CYP4V2*	NM_207352.4	rs199476204	c.1348C>T	p.Gln450Ter	Substitution	Nonsense	Homozygous	AR		Pathogenic	PVS1	_	_	_	_	_
**IRD-15**	Croatia	41	38	RP	*PDE6B*	NM_000283.4	rs370898371	c.1107+3A>G	IVS8+3A>G	Substitution	Intron Variant	Compound Het	AR	Gene Panel Testing	Likely Pathogenic	PS4	_		_	_	
							rs1737315492	c.1859A>G	p.His620Arg	Substitution	Missense		AR		Likely Pathogenic	PS4	_	Disease causing	_	_	
**IRD-18**	Qatar	25	24	RP	*PDE6B*	NM_000283.4	rs751859807	c.1655G>A	p.Arg552Gln	Substitution	Missense	Homozygous	AR	Gene Panel testing	Pathogenic	PS4	_	_			
**IRD-10**	Qatar	15	9	RP	*PDE6C*	NM_006204.4	rs1057518244	c.724-1G>T	IVS3-1G>T (in intron3)	Substitution	Splice Acceptor	Homozygous	AR	WES Plus	Pathogenic	PVS1	_	_			
**IRD-19**	Palestine	21	17	RP	*RDH12*	NM_152443.3	rs1594867597	c.821T>C	p.Leu274Pro	Substitution	Missense	Homozygous	AR	WES Plus	Pathogenic	PS4	_	_	Israel	RP, LCA	[17]
**IRD-31**	Palestine	6	2.5	Uncategorized Retinal Dystrophy	*RDH12*	NM_152443.3	rs1594867597	c.821T>C	p.Leu274Pro	Substitution	Missense	Homozygous	AR	WES Plus	Pathogenic	PS4	_	_	Israel	RP, LCA	[17]
**IRD-24**	Qatar	50	49	RP	*KIZ*	NM_018474.6	rs775124094	c.247C>T	p.Arg83Ter	Substitution	Nonsense	Homozygous	AR	Gene Panel testing	Pathogenic	PM3	_	_	_	_	_
**IRD-29**	Qatar	37	37	RP	*RPGR*	NM_001034853.2	rs1186795749	c.3092del	p.Glu1031Glyfs*58	Deletion	Frameshift	Hemizygous	XLR	Gene Panel testing	Pathogenic	PM3	_	Disease causing	Denmark	RP	[18]
**IRD-30**	Syria	14	12	Macular dystrophy	*AIPL1*	NM_014336.5	rs62637014	c.834G>A	p.Trp278Ter	Substitution	Nonsense	Homozygous	AR	WES Plus	Pathogenic	PM3	_	Disease causing	Romania	LCA	[19]
**IRD-32**	Qatar	44	41	RP	*GNAT2*	NM_001377295.2	rs1553226581	c.720+5G>C	IVS6+5G>C	Substitution	Splicing site	Homozygous	AR	WES Plus	Likely Pathogenic	PM3	_	_	_	_	_
**IRD-44**	Qatar	16	11	Achromatopsia	*GNAT2*	NM_001377295.2	rs1553226581	c.720+5G>C	IVS6+5G>C	Substitution	Splicing site	Homozygous	AR	WES Plus	Likely Pathogenic	PM3	_	_	_	_	_
**IRD-33**	Pakistan	9	6	LCA	*NMNAT1*	NM_022787.4	rs201994921	c.634G>A	p.Val212Met	Substitution	Missense	Compound Heterozygous	AR	Familial Targeted Testing	Likely Pathogenic	PM1	_	_	_	LCA	_
							_	chr1: 10035650_10035833	_	Deletion	Deletion		AR		Likely Pathogenic	_	_	_	_	_	_
**IRD-35**	Lebanon	7	6	Rod & Rod-Cone dystrophy	*RPGRIP1*	NM_020366.4	_	c.3278dupC	p.Gln1094Thrfs*6	Duplication	Frameshift	Compound Heterozygous	AR	WES Plus	Likely Pathogenic	PVS1	_	Disease causing	_	_	_
							rs1371805993	c.2935C>T	p.Gln979Ter	substitution	Missense		AR		Pathogenic	PVS1	_	_	Israel	RP	[17]
**IRD-14**	Qatar	5	2	Uncategorized Retinal Dystrophy	*RPGRIP1*	NM_020366.4	rs61751266	c.1107delA	p.Glu370AsnfsX5	Deletion	Frameshift	Homozygous	AR	WES	Pathogenic	PM3	_	_	Saudi Arabia	Cone-Rod Dystrophy, LCA	[20]
**IRD-42**	Lebanon	27	27	RP	*CFAP418*	NM_177965.4	_	c.478dupA	p.Met160Asnfs*25	Duplication	Frameshift	Homozygous	AR	WES Plus	Likely Pathogenic	PVS1	_	Disease causing	_	_	_
**IRD-46**	Qatar	61	61	Macular dystrophy	*PRPH2*	NM_000322.5	rs1799986489	c.936del	p.Pro313Argfs*11	Deletion	Frameshift	Heterozygous	AD	Familial Targeted Testing	Likely Pathogenic	PVS1	_	Disease causing	_	_	_
**IRD-12**	Qatar	7	7	RP	*CNGA3*	NM_001298.3	rs104893613	c.847C>T	p.Arg283Trp	Substitution	Missense	Homozygous	AR	WES	Pathogenic	PM3	_	_	_	_	_
**IRD-41**	Egypt	13	9	Cone-Rod dystrophy	*CFAP410*	NM_004928.3	rs771024688	c.209G>A	p.Arg70Gln	Substitution	Missense	Homozygous	AR	WES Plus Trio	Likely Pathogenic	PM2	_	Disease causing	_	_	_
**IRDs-45**	Qatar	54 years	50 years	RP	*ABCA4*	NM_000350.3	rs752850266	c.6218G>C	p.Gly2073Ala	Substitution	Missense	Homozygous	AR	WES Plus	Likely Pathogenic	PM1	_	Disease causing	_	_	_
**IRDs-28**	United Arab Emirates	10 years	6 years	Macular dystrophy	*CRB1*	NM_201253.3	rs1571522690	c.1313G>A	p.Cys438Tyr	Substitution	Missense	Homozygous	AR	Gene Panel testing	Likely Pathogenic	PM1	_	Disease causing	_	_	_
**IRDs-49**	Egypt	12 years	3 years	LCA	*RPGRIP1*	NM_020366.4	_	c.105dupA	p.Pro36Thrfs*35	Duplication	Frameshift	Homozygous	AR	Gene Panel testing	Likely Pathogenic	PVS1	_	Disease causing	_	_	_
**IRD-23**	Qatar	48	46	RP	*PRCD*	NM_001077620.3	rs757471313	c.74C>T	p.Pro25Leu	Substitution	Missense	Homozygous	AR	WES Plus Trio	Likely Pathogenic	PM3	_	Disease causing	_	_	_
**IRD-11**	Qatar	21	20	RP	*MERTK*	NM_006343.3	_	c.2020A>G	p.Met674Val	Substitution	Missense	Homozygous	AR	WES Trio	Likely Pathogenic	PM3	_	Disease causing	_	_	_
**IRD-39**	Yemen	3	1.5	LCA	*GUCY2D*	NM_000180.4	_	c.2213_2215del	p.Glu738del	Deletion	Frameshift	Homozygous	AR	WES Trio	Likely Pathogenic	PS4	_	Disease causing	_	_	_
**IRD-13**	Qatar	48	40	RP	*MERTK*	NM_006343.3	_	c.2020A>G	p.Met674Val	Substitution	Missense	Homozygous	AR	WES	Likely Pathogenic	PM3	_	Disease causing	_	_	_
							rs141361084	c.2435A>C	p.Tyr812Ser	Substitution	Missense	Homozygous	AR		** Variant of uncertain significance	PM2	Warm	Disease causing	_	_	_

* Patient’s age at the time of data collection. ** Secondary finding: one variant of uncertain significance (VUS) identified in patient IRD-13; this does not affect the case classification outcome. AD: Autosomal dominant, AR: Autosomal recessive, XLR: X-linked recessive, WES: Whole exome sequencing, PVS1: Very strong evidence of pathogenicity, PS3: Strong evidence of pathogenicity, PM1-PM6: Moderate strength evidence of pathogenicity, RP: Retinitis Pigmentosa, LCA: Leber Congenital Amaurosis.

## Data Availability

The data from the Department of Adult and Pediatric Medical Genetics database is not publicly available to ensure the privacy and confidentiality of the patients involved in the study. The data are available from the Department of Adult and Pediatric Medical Genetics; however, restrictions apply to their availability, as they were used under license for the current study and are not publicly accessible.

## Supplementary Materials

The following supporting information can be downloaded at https://www.mdpi.com/article/10.3390/genes16121415/s1: Figure S1: Pedigrees showing variants segregating with the disease in cases re-evaluated as likely to be solved after family segregation studies. Generated by CeGaT pedigree chart designer. (W: wild type, M: mutant), Figure S2: CRB1 predicted protein model using I-Tasser, Figure S3: CRB1(2) predicted protein model using I-Tasser, Figure S4: CFAP418 predicted protein model using I-Tasser, Figure S5: RPGRIP1 predicted protein model using I-Tasser, Figure S6: RPGRIP1(2) predicted protein model using I-Tasser, Figure S7: GUCY2D predicted protein model using I-Tasser, Table S1: Overview of Gene Panels Used in the Diagnosis of Inherited Retinal Dystrophies (IRD), Table S2: Identified variants in cases re-evaluated as likely to be solved after family segregation studies, Table S3: Identified variants in the uncertain cases, Table S4: Identified Shared Genetic Variants Across Diverse Populations [3,17,18,19,20,21,22,29,41,53], Table S5: Novel Genetic Variants Identified in Our Patient Population, Table S6: Validating wild-type protein structures using different validation tools, Table S7: The diagnostic yield of different genetic tests.

## Abbreviations

IRD	Inherited Retinal Dystrophies
LCA	Leber Congenital Amaurosis
RP	Retinitis Pigmentosa
HMC	Hamad Medical Corporation
WES	Whole-exome sequencing
WES Plus	Whole-exome sequencing and mitochondrial genome testing
WGS	Whole genome sequencing
NGS	Next-generation sequencing
ACMG	American College of Medical Genetics and Genomics
VUS	Variant of uncertain significance
AD	Autosomal dominant
AR	Autosomal recessive
AD/AR	Autosomal dominant & Autosomal recessive
XL	X-linked
AVMD	Adult-onset vitelliform macular dystrophy
AAV	Adeno-associated virus
