# Supplementary material for: Exploring the Genetic Causes of Nonsyndromic Retinal Dystrophies in Qatar
Source: Genes (Basel). 2025 Nov 27;16(12):1415. doi: 10.3390/genes16121415 (PMC12733087; doi:10.3390/genes16121415)
Supplement: Supplementary file 1 [file genes-16-01415-s001.zip › Supplementary Table Final.pdf]

Supplemental Table S1. Overview of Gene Panels Used in the Diagnosis of Inherited Retinal Dystrophies (IRD)

Gene Panel	Number of Genes Tested	Genes Tested	Analysis Method
Congenital Stationary Night Blindness (CSNB)	12	*CABP4, CACNA1F, CHM, GNAT1, GRM6, NYX, PDE6B, RDH5, RHO, RPE65, SAG, TRPM1*	Sequencing, Deletion/Duplication Analysis
Cone-Rod Dystrophies Panel	31	*ABCA4, ADAM9, AIPL1, BEST1, C8orf37, CABP4, CACNA1F, CDH3, CDHR1, CEP290, CERKL, CNGA3, CNGB3, CRX, DRAM2, ELOVL4, GUCA1A, GUCY2D, PAX6, PITPNM3, POC1B, PROM1, RAB28, RAX2 (QRX), RDH5, RD (PRPH2), RIMS1, RPGR, RPGRIP1, SEMA4A, TTLL5*	Sequencing Analysis
Retinal Dystrophy Xpanded Panel	~780	Comprehensive coverage of about 780 genes	Sequencing Analysis, Optional "Trio" Approach

Supplemental Table S2.. Identified variants in cases re-evaluated as likely to be solved after family segregation studies.

PATIENT ID	Country of Origin	Age (Years)*	Age of Diagnosis	Patient Phenotype	Gene	Gene (NM Number)	RSID	Variant (cDNA)	Variant (Protein)	Variant Type	Variant Impact	Zygosity	Pattern of Inheritance	Test Performed	ACMG	ACMG Highest Pathogenicity Evidence	VUS Subclassification	Mutation Taster	SIFT	PolyPhen	CADD Score	GnomAD Allele Frequency	Populations with Allele Detected	Mother	Father	Affected Siblings	Healthy Siblings	Justification	Reports from Other Populations/Ethnicities	Reported Phenotype	References
**IRD-7**	Pakistan	15	14	RP	*PDE6B*	NM_000283.4	rs771338607	c.2407A>G	p.Asn803Asp	Substitution	Missense	Homozygous	AR	WES Plus Trio	Variant of uncertain significance	PM2	Warm	_	Deleterious	Probably damaging	28	Heterozygous - 0.00000398	South Asian	Heterozygous	Heterozygous	No affected siblings	Heterozygous in 1 brother, not detected in 2 sisters	Most likely associated with RP in this patient as the variant homozygosity is segregating with the disease in the family	_	_	_
**IRD-40**	Egypt	20	14	Cone-Rod dystrophy	*ABCA4*	NM_000350.3	rs745512565	c.4753C>T	p.Arg1585Trp	Substitution	Missense	Homozygous	AR	WES Trio	Variant of uncertain significance	PM1	Warm	Disease causing	Deleterious	Benign	23	Heterozygous - 0.000006579	African/African American	Heterozygous	Heterozygous	No affected siblings	Heterozygous in 1 sister, not detected in 1 brother	Most likely associated with Cone-Rod dystrophy in this patient as the variant homozygosity is segregating with the disease	_	_	_
**IRD-48**	Qatar	5	2	Cone-Rod dystrophy	*CNNM4*	NM_020184.4	_	c.509T>C	p.Leu170Pro	Substitution	Missense	Homozygous	AR	Familial targeted testing	Variant of uncertain significance	PM2	Tepid	Disease causing	Uncertain	Probably damaging	26	_	_	Heterozygous	Heterozygous	No affected siblings	Heterozygous in 1 unaffected sister	Most likely associated with Cone-Rod dystrophy in this patient as the variant homozygosity is segregating with the disease	Qatar, UAE	Cone-Rod Dystrophy, Jalili	[41,3]
**IRD-16**	Qatar	38	33	RP	*PCDH15*	NM_001384140.1	rs568865061	c.2897G>C	p.Arg966Thr	Substitution	Missense	Compound Heterozygous	AR	WES Plus Proband	Variant of uncertain significance	BS1	Ice cold	_	Deleterious	Probably damaging	21	Heterozygous - 0.00001972	South Asian	Not detected	Heterozygous	No affected siblings	Not detected in 2 siblings	Most likely associated with RP in this patient as the variant compound heterozygosity is segregating with the disease	_	_	_
							rs750302536	c.131T>C	p.Val44Ala	Substitution	Missense		AR		Variant of uncertain significance	PM2	Warm	Disease causing	Deleterious	Probably damaging	25	Heterozygous - 0.00005263	European (non-finnish) Lationo/Admixed American South Asian	Heterozygous	Not detected	No affected siblings	Not detected in 2 siblings		_	_	_
**IRD-37**	Pakistan	12	8	Cone-Rod dystrophy	*GRM6*	NM_000843.4	_	c.281G>C	p.Arg94Pro	Substitution	Missense	Homozygous	AR	WES Plus Trio	Variant of uncertain significance	PM2	Hot	Disease causing	Uncertain	Probably damaging	24	_	_	Heterozygous	Heterozygous	No affected siblings	Not detected in 2 sisters	Most likely associated with Cone-Rod dystrophy in this patient as the variant homozygosity is segregating with the disease	_	_	_

* Patient's age at the time of data collection. AD: Autosomal dominant, AR: Autosomal recessive, AD/AR: Autosomal dominant & Autosomal recessive, XL: X-linked, PVS1: Very strong evidence of pathogenicity, PS3: Strong evidence of pathogenicity, PM1-PM6: Moderate strength evidence of pathogenicity, RP: Retinitis Pigmentosa, LCA: Leber Congenital Amaurosis.

Supplemental Table S3. Identified variants in the uncertain cases

Patient ID	Country of origin	Age*	Age of diagnosis	Phenotype	Gene	RSID	Variant (c.DNA)	Variant (protein)	Variant type	Variant Impact	Zygosity	Pattern of Inheritance	Test Performed	ACMG	ACMG Highest Pathogenicity Evidence	VUS Subclassification	MutationTaster	SIFT	PolyPhen	CADD Score	Reports from other populations/ethnicities	Reported Phenotype	References
IRD-3	Qatar	46 years	43 years	RP	*RHO*	–	c.697-3C>A	IVS3-3C>A	Substitution	Splicing site	Homozygous	AR	WES Plus	Variant of uncertain significance	PM2	Warm	–	–	–	–	–	–	–
IRD-17	Qatar	3 years	2 years	RP	*PDE6A*	rs374847529	c.103G>A	p.Asp35Asn	Substitution	Missense	Homozygous	AR	WES	Variant of uncertain significance	PM1	Warm	Disease causing	Tolerated	Benign	18	–	–	–
					GPC4	rs1412463359	c.156C>G	p.lle52Met	Substitution	Missense	Hemizygous	XL		Variant of uncertain significance	PM2	Cool	Disease causing	Deleterious	Possibly damaging	23	–	–	–
IRD-34	Pakistan	56 years	53 years	Stargardt disease	*ABCA4*	rs1801466	c.5603A>T	p.Asn1868IIe	Substitution	Missense	Heterozygous	AD	WES	Benign	PP2	–	Polymorphism	Deleterious	Benign	20	–	–	–
					DRAM2	rs772262465	c.246T>G	p.Ser82Arg	Substitution	Missense	Homozygous	AR		Variant of uncertain significance	PM2	Warm	Disease causing	Tolerated	Benign	22	–	–	–
IRD-38	Qatar	14 years	8 years	Congenital stationary night blindness	*EYS*	rs199740930	c.2137+1G>A	IVS13+1G>A	Substitution	Splice site donor	Compound Heterozygous	AR	WES Plus	Pathogenic	PM3	–	–	–	–	–	–	–	–
						rs1383398602	c.3709G>C	p.Gly1237Arg	Substitution	Missense		AR		Variant of uncertain significance	PM2	Cool	–	Deleterious	Benign	15	–	–	–
IRD-43	Palestine	13 years	6 years	Cone dystrophy	*GUCA1B*	rs1554186885	c.593C>G	p.Ala198Gly	Substitution	Missense	Heterozygous	AD	WES	Variant of uncertain significance	PM2	Cool	Disease causing	Deleterious	–	23	–	–	–
IRD-20	Egypt	14	12	Cone dystrophy	*KCNV2*	–	9p24.2(2684449-2766856)x1	–	Copy number variation	Copy number variation	Compound Heterozygous	AR	WES	Pathogenic	–	–	–	–	–	–	–	–	–
						rs1819788466	c.757C>G	p.Pro253Ala	Substitution	Missense		AR		Variant of uncertain significance	PM2	Warm	Disease causing	Deleterious	Possibly damaging	25	–	–	–
IRD-36	Qatar	10	6	Cone-Rod dystrophy	*CRB1*	–	c.3613G>T	p.Gly1205Ter	Substitution	Nonsense	Compound Heterozygous	AR	WES Plus	Likely Pathogenic	PVS1	–	–	–	–	–	–	–	–
						–	c.4211G>C	p.Arg1404Thr	substitution	Missense		AR		Variant of uncertain significance	PM2	Warm	–	–	Robably damaging	24	–	–	–

*Age at data collection. AD: Autosomal dominant, AR: Autosomal recessive, AD/AR: Autosomal dominant & Autosomal recessive, XL: X-linked, PVS1: Very strong evidence of pathogenicity, PS3: Strong evidence of pathogenicity, PM1-PM6: Moderate strength evidence of pathogenicity, RP: Retinitis Pigmentosa, LCA: Leber Congenital Amaurosis.

Supplemental Table S4. Identified Shared Genetic Variants Across Diverse Populations

Gene	*RSID*	*Variant (c.DNA)*	*Variant (protein)*	*Variant type*	*Variant Impact*	*Zygosity*	*Pattern of Inheritance*	*ACMG*	*ACMG Highest Pathogenicity Evidence*	*VUS Subclassification*	*Mutationtaster*	*ClinVar classification*	*ClinVar phenotype*	*GnomAD Allele Frequency*	*Populations with Allele Detected*	*GME Allele Frequency*	*PATIENT ID*	*Country of origin*	*Age* (years)*	*Age of diagnosis (years)*	*Phenotype*	*reports from other populations/ethnicities*	*Reported Phenotype*	*References*
GUCY2D	rs763890649	c.1040_1041delTT	p.Phe347TrpfsX5	Deletion	Frameshift	Homozygous	AR	Pathogenic	_	_	disease causing	Likely Pathogenic	LCA	Heterozygous -0.000004164	_	_	IRD-1	Qatar	2 years	3 months	RP	_	_	_
	rs138836357	c.1093C>T	p.Arg365Trp	Substitution	Missense	Homozygous	AR	Benign	_	_	polymorphism	Conflicting interpretations of Pathity	LCA	Heterozygous - 0.0007357	Middle Eastren Lationo/Admixed American African/African American European (non-finnish) European (finnish) Ashkenazi Jewish South Asian	0.002014099	IRD-30	Syria	14 years	12 years	Macular dystrophy	_	_	_
MERTK	rs886039422	c.2214delT	p.Cys738TrpfsX32	Deletion	Frameshift	Homozygous	AR	Pathogenic	_	_	disease causing	Pathogenic	RP (AR)	_	_	_	IRD-1	Qatar	2 years	3 months	RP	Saudi Arabia, United Arab Emirate	RP & Rod cone dystrophy	[22,41]
																	IRD-21	Qatar	34 years	28 years	RP			
	_	c.2020A>G	p.Met674Val	Substitution	Missense	Homozygous	AR	Likely Pathogenic	_	_	disease causing	Likely Pathogenic	_	-	-	-	IRD-11	Qatar	21 years	20 years	RP	_	_	_
						Homozygous											IRD-13	Qatar	48 years	40 years	RP			
	rs141361084	c.2435A>C	p.Tyr812Ser	Substitution	Missense	Homozygous	AR	Variant of uncertain significance	PM2	Warm	disease causing	Variant of uncertain significance	RP	Heterozygous -0.00007884	Middle Eastren Lationo/Admixed American African/African American European (non-finnish)	0.003021148								
GRM6	rs752205220	c.1478G>A	p.Trp493Ter	Substitution	Nonsense	Homozygous	AR	Pathogenic	_	_	disease causing	-	-	Heterozygous - 0.000007983	Lationo/Admixed American	_	IRD-2	Qatar	3 years	3 years	Congenital stationary night blindness	_	_	_
																	IRD-6	Qatar	6 years	6 years	Congenital stationary night blindness			
	_	c.281G>C	p.Arg94Pro	Substitution	Missense	Homozygous	AR	Variant of uncertain significance	PM1	Hot	disease causing	Variant of uncertain significance	_	-	-	-	IRD-37	Pakistan	12 years	8 years	RP	_	_	_
CABP4	rs786205852	c.81_82insA	p.Pro28ThrfsX4.	Insertion	Frameshift	Homozygous	AR	Pathogenic	_	_	disease causing	Pathogenic	_	-	-	-	IRD-4	Qatar	4 years	6 months	Uncategorized Retinal dystrophy	Saudi Arabia	segregated with congenital retinal dysfunction in 11 affected individuals (aged 2–26 years) from four consanguineous families	[21]
ABCA4	_	c.5584G>C	p.Gly1862Arg	Substitution	Missense	Homozygous	AR	Pathogenic	PS4	_	disease causing	Pathogenic	_	-	-	-	IRD-8	Qatar	26 years	23 years	Stargardt disease	China	Stargardt disease	[53]
	rs61748556	c.1609C>T	p.Arg537Cys	Substitution	Missense	Heterozygous	AR	Likely Pathogenic	PS4	_	_	Pathogenic/Likely pathogenic	Retinal dystrophy	Heterozygous -0.00002387	African/African American European (non-finnish) South Asain European (finnish	_	IRD-9	Qatar	14 years	11 years	Macular dystrophy	Germany	Stargardt disease	[29]
	rs61750155	c.4793C>A	p.Ala1598Asp	Substitution	Missense	Homozygous	AR	Pathogenic	PM3	_	_	Pathogenic/Likely pathogenic	Retinal dystrophy	Heterozygous -0.00002631	Lationo/Admixed American European (non-finnish)	0.001007049	IRD-25	Yemen	20 years	18 years	Stargardt disease	Germany	Stargardt disease	[19]

	rs1800553	c.5882G>A	p.Gly1961Glu	Substitution	Missense	Heterozygous	AR	Likely Pathogenic	PS3	–	–	Pathogenic	Complex Retinal dystrophy	–	–	–	IRD-9	Qatar	14 years	11 years	Macular dystrophy			
																	IRD-47	Qatar	69 years	64 years	RP	China, Spain, United Arab Emirates, and Italy	Stargardt disease	[17,18,53]
																	IRD-22	Qatar	56 years	53 years	RP			
	rs1801466	c.5603A>T	p.Asn1868Ile	Substitution	Missense	Heterozygous	AD	Benign	PP2	–	polymorphism	Conflicting interpretations of pathogenicity	Complex Retinal dystrophy	Heterozygous/ Homozygous - 0.04042	Lationo/Admixed American African/African American European (non-finnish) Amish Middle Eastren Askenazi Jewish European (finnish) South Asian	0.039314516	IRD-34	Pakistan	56 years	53 years	Stargardt disease	Germany	Stargardt disease	[29]
	rs745512565	c.4753C>T	p.Arg1585Trp	Substitution	Missense	Homozygous	AR	Variant of uncertain significance	PM1	Warm	disease causing	Variant of uncertain significance	–	Heterozygous -0.000006579	African/African American	–	IRD-40	Egypt	20 years	14 years	Cone-Rod dystrophy	–	–	–
	rs752850266	c.6218G>C	p.Gly2073Ala	Substitution	Missense	Homozygous	AR	Variant of uncertain significance	PM1	Hot	disease causing	Variant of uncertain significance	–	Heterozygous - 0.00003942	African/African American European (non-finnish)	–	IRD-45	Qatar	54 years	50 years	RP	–	–	–
CRX	rs771736389	c.128G>A	p.Arg43His	Substitution	Missense	Heterozygous	AD	Variant of uncertain significance	PM1	Hot	disease causing	Pathogenic/Likely pathogenic	Cone-rod dystrophy LCA	Heterozygous -0.000006574	European (non-finnish)	–	IRD-9	Qatar	14 years	11 years	Macular dystrophy	–	–	–
PDE6B	rs370898371	c.1107+3A>G	IVS8+3A>G	Substitution	Splice site	compound Heterozygous	AR	Likely Pathogenic	PS4	–	–	Conflicting interpretations of pathogenicity	RP	Heterozygous -0.00002627	European (non-finnish)	–	IRD-15	Croatia	41 years	3 years	RP	–	–	–
	rs1737315492	c.1859A>G	p.His620Arg	Substitution	Missense		AR	Likely Pathogenic	PS4	Hot	disease causing	Variant of uncertain significance	RP	-	-	–						–	–	–
	rs751859807	c.1655G>A	p.Arg552Gln	Substitution	Missense	Homozygous	AR	Pathogenic	PS4	–	–	Pathogenic/Likely pathogenic	–	Heterozygous-0.00001972	East Asian European (non-finnish) African/African American	–	IRD-18	Qatar	25 years	24 years	RP	–	–	–
	rs771338607	c.2407A>G	p.Asn803Asp	Substitution	Missense	Homozygous	AR	Variant of uncertain significance	PM2	Warm	–	Variant of uncertain significance	RP	Heterozygous -0.00000398	South Asian	–	IRD-7	Pakistan	15 years	14 years	RP	–	–	–
PDE6C	rs1057518244	c.724-1G>T	IVS3-1G>T (in intron3)	Substitution	Splice site	Homozygous	AR	Pathogenic	PM3	–	–	Likely pathogenic	–	-	-	-	IRD-10	Qatar	15 years	9 years	RP	–	–	–
RDH12	rs1594867597	c.821T>C	p.Leu274Pro	Substitution	Missense	Homozygous	AR	Pathogenic	PVS1	–	–	Pathogenic	RP	-	-	-	IRD-19	Palestine	21 years	17 years	RP	Israel	RP, LCA	[20]

																	IRD-31	Palestine	6 years	2.5 years	Uncategorized Retinal dystrophy			
KCNV2	–	9p24.2(2684449-2766856)x1	–	copy number variation	copy number variation	compound Heterozygous	AR	Pathogenic	__	–	–	–	–	–	–	–	IRD-20	Egypt	14 years	12 years	Cone dystrophy	–	–	–
	rs1819788466	c.757C>G	p.Pro253Ala	Substitution	Missense		AR	Variant of uncertain significance	PM2	Warm	disease causing	Variant of uncertain significance	_	-	-	-						_	_	_
KIZ	rs775124094	c.247C>T	p.Arg83Ter	Substitution	Nonsense	Homozygous	AR	Pathogenic	PM3	_	_	Pathogenic/Likely pathogenic	Retinal dystrophy	Heterozygous -0.00003287	African/African American European (non-finnish)	-	IRD-24	Qatar	50 years	49 years	RP	_	_	_
RPGR	rs1186795749	c.3092del	p.Glu1031Glyfs*58	Deletion	Frameshift	Hemizygous	XLR	Pathogenic	PM3	_	disease causing	Pathogenic	Complex Retinal dystrophy	-	-	-	IRD-29	Qatar	37 years	37 years	RP	Denmark	RP	[17]
AIPL1	rs62637014	c.834G>A	p.Trp278Ter	Substitution	Nonsense	Homozygous	AR	Pathogenic	PM3	Warm	disease causing	Pathogenic	LCA	Heterozygous -0.0003291	Lationo/Admixed American African/African American European (non-finnish) South Asian	0.000503525	IRD-30	Syria	14 years	12 years	Macular dystrophy	Romania	LCA	[18]
GNAT2	rs1553226581	c.720+5G>C	IVS6+5G>C	Substitution	Splice site	Homozygous	AR	Likely Pathogenic	PM3	_	_	Likely pathogenic	_	-	-	-	IRD-32	Qatar	44 years	41 years	RP	_	_	_
																	IRD-44	Qatar	16 years	11 years	Achromatopsia	_	_	_
NMNAT1	rs201994921	c.634G>A	p.Val212Met	Substitution	Missense	compound Heterozygous	AR	Likely Pathogenic	PM1	_	_	Conflicting interpretations of pathogenicity	LCA	Heterozygous -0.00003286	African/African American European (non-finnish) European (finnish)	_	IRD-33	Pakistan	9 years	6 years	Generalized Retinal dystrophies	_	_	_
	_	chr1: 10035650_10035833	_	Deletion	Copy number variation		AR	Likely Pathogenic	_	_	_	_	-	-	-	_						_	_	_
RPGRIP1	rs1371805993	c.2935C>T	p.Gln979Ter	Substitution	Missense	Heterozygous	AR	Pathogenic	PVS1	_	_	Pathogenic	LCA Cone-rod dystrophy, RP	_	_	_	IRD-35	Lebanon	7 years	6 years	Rod-Cone dystrophy	Israel	RP	[20]
	rs61751266	c.1107delA	p.Glu370AsnfsX5	Deletion	Frameshift	Homozygous	AR	Pathogenic	_	_	_	Pathogenic	LCA	_	_	_	IRD-14	Qatar	5 years	2 years	Uncategorized Retinal dystrophy	Saudi Arabia	Cone-Rod Dystrophy, LCA	[3]
CRB1	rs1571522690	c.1313G>A	p.Cys438Tyr	Substitution	Missense	Homozygous	AR	Likely Pathogenic	PM1	_	disease causing	Variant of uncertain significance	LCA	-	-	-	IRD-28	United arab emirates	10 years	6 years	Macular dystrophy	_	_	_
PRPH2	rs1799986489	c.936del	p.Pro313Argfs*11	Deletion	Frameshift	Heterozygous	AD	Likely Pathogenic	PVS1	_	disease causing	Conflicting interpretations of pathogenicity	Complex Retinal dystrophy	-	-	-	IRD-46	Qatar	61 years	61 years	Macular dystrophy	_	_	_

CYP4V2	rs199476204	c.1348C>T	p.Gln450Ter	Substitution	Nonsense	Homozygous	AR	Pathogenic	PVS1	_	_	Pathogenic	Bietti Crystalline Dystrophy	Heterozygous -0.000006576	European (non-finnish)	-	IRD-47	Qatar	69 years	64 years	RP	_	_	_
RHO	_	c.697-3C>A	IVS3-3C>A	Substitution	Splice site	Homozygous	AR	Variant of uncertain significance	PM2	Warm	_	Variant of uncertain significance	_	_	_	_	IRD-3	Qatar	46 years	43 years	RP	_	_	_
PCDH15	rs568865061	c.2897G>C	p.Arg966Thr	Substitution	Missense	compound Heterozygous	AR	Variant of uncertain significance	BS1	Ice Cold	_	Variant of uncertain significance	_	Heterozygous -0.00001972	South Asian	_	IRD-16	Qatar	38 years	33 years	RP	_	_	_
	rs750302536	c.131T>C	p.Val44Ala	Substitution	Missense		AR	Variant of uncertain significance	PM2	Warm	disease causing	Conflicting interpretations of pathogenicity	Usher syndrome type 1F, Autosomal recessive nonsyndromic HL	Heterozygous -0.00005263	European (non-finnish) Lationo/Admixed American South Asian	_						_	_	_
PDE6A	rs374847529	c.103G>A	p.Asp35Asn	Substitution	Missense	Homozygous	AR	Variant of uncertain significance	PM1	Warm	disease causing	Variant of uncertain significance	RP	Heterozygous -0.0001251	European (non-finnish) East Asian Lationo/Admixed American African/African American	0.0025176233635448137	IRD-17	Qatar	3 years	2 years	RP	_	_	_
GPC4	rs1412463359	c.156C>G	p.lle52Met	Substitution	Missense	Hemizygous	XL	Variant of uncertain significance	PM2	Cool	disease causing	-	-	Heterozygous /Hemizygous - 0.00001102	European (non-finnish)	_						_	_	_
PRCD	rs757471313	c.74C>T	p.Pro25Leu	Substitution	Missense	Homozygous	AR	Likely Pathogenic	PM3	_	disease causing	Variant of uncertain significance	_	Heterozygous -0.00001633	European (non-finnish) East Asian	_	IRD-23	Qatar	48 years	46 years	RP	_	_	_
DRAM2	rs772262465	c.246T>G	p.Ser82Arg	Substitution	Missense	Homozygous	AR	Variant of uncertain significance	PM2	Warm	polumorphism	Variant of uncertain significance	_	Heterozygous -0.00001195	South Asian	_	IRD-34	Pakistan	56 years	53 years	Stargardt disease	_	_	_
EYS	rs199740930	c.2137+1G>A	IVS13+1G>A	Substitution	Splice site	compound Heterozygous	AR	Pathogenic	PM3	_	_	Conflicting interpretations of pathogenicity	RP	Heterozygous-0.000006409	Ashkenazi Jewish Middle Eastren South Asian European (non-finnish) Lationo/Admixed American African/African American	_	IRD-38	Qatar	14 years	8 years	Congenital stationary night blindness	Denmark	RP	[17]
	rs1383398602	c.3709G>C	p.Gly1237Arg	Substitution	Missense		AR	Variant of uncertain significance	PM2	Tepid	_	Variant of uncertain significance	_	Heterozygous-0.0003553	South Asian	_						-	_	
CFAP410	rs771024688	c.209G>A	p.Arg70Gln	Substitution	Missense	Homozygous	AR	Variant of uncertain significance	PM1	Warm	disease causing	Variant of uncertain significance	Retinal dystrophy	Heterozygous -0.00001314	South Asian European (non-finnish)	_	IRD-41	Egypt	13 years	9 years	Cone-Rod dystrophy	_	_	_
GUCA1B	rs1554186885	c.593C>G	p.Ala198Gly	Substitution	Missense	Heterozygous	AD	Variant of uncertain significance	PM2	Cool	disease causing	Variant of uncertain significance	_	-	-	-	IRD-43	Palestine	13 years	6 years	Cone dystrophy	_	_	_

TUB	rs575184271	c.1357_1360delAGAG	p.Arg453SerfsX13	Deletion	Frameshift	Homozygous	Unknown	_	_	_	disease causing	-	-	Heterozygous -0.000003977	European (finnish)	–						–	–	–
CNGA3	*rs104893613*	c.847C>T	p.Arg283Trp	Substitution	Missense	Homozygous	AR	Pathogenic	PM3	_	_	Pathogenic/Likely pathogenic	Achromatopsia	Heterozygous -0.00001971	European (non-finnish) African/African American	–	IRD-12	Qatar	7 years	7 years	Cone dystrophy	–	–	–
CNNM4	_	c.509T>C	p.Leu170Pro	Substitution	Missense	Homozygous	AR	Variant of uncertain significance	PM2	Tepid	disease causing	_	–	–	–	–	IRD-48	Qatar	5 years	2 years	Cone-Rod dystrophy	Qatar, United Arab Emirates	Cone-Rod Dystrophy, Jalili	[3,22]

*Age at data collection. AD: Autosomal dominant, AR: Autosomal recessive, AD/AR: Autosomal dominant & Autosomal recessive, XL: X-linked, PVS1: Very strong evidence of pathogenicity, PS3: Strong evidence of pathogenicity, PM1-PM6: Moderate strength evidence of pathogenicity, RP: Retinitis Pigmentosa, LCA: Leber Congenital Amaurosis.

Supplemental Table S5. Novel Genetic Variants Identified in Our Patient Population

Gene	*RSID*	*Variant (c.DNA)*	*Variant (protein)*	*Variant type*	*Variant Impact*	*Zygosity*	*Pattern of Inheritance*	*Test Performed*	*ACMG*	*ACMG Highest Pathogenicity Evidence*	*Mutation taster*	*PATIENT ID*	*Country of origin*	*Age**	*Age of diagnosis*	*Phenotype*
GUCY2D	_	c.2213_2215del	p.Glu738del	Deletion	Frameshift	Homozygous	AR	WES	Likely Pathogenic	PS4	disease causing	IRD-39	Yemen	3 years	1.5 years	LCA
RPGRIP1	_	c.3278dupC	p.Gln1094Thrfs*6	Duplication	Frameshift	compound Heterozygous	AR	WES Plus	Likely Pathogenic	PVS1	disease causing	IRD-35	lebanon	7 years	6 years	Rod-Cone dystrophy
	_	c.105dupA	p.Pro36Thrfs*35	Duplication	Frameshift	Homozygous	AR	Panel	Likely Pathogenic	PVS1	disease causing	IRD-49	Egypt	12 years	3 years	LCA
CRB1	_	c.3613G>T	p.Gly1205Ter	Substitution	Nonsense	compound Heterozygous	AR	WES Plus	Likely Pathogenic	PVS1	disease causing	IRD-36	Qatar	10 years	6 years	Cone-Rod dystrophy
	_	c.4211G>C	p.Arg1404Thr	Substitution	Missense		AR		Variant of uncertain significance	PM2	disease causing					
CFAP418	_	c.478dupA	p.Met160Asnfs*25	Duplication	Frameshift	Homozygous	AR	WES Plus	Likely Pathogenic	PVS1	disease causing	IRD-42	lebanon	27 years	27 years	RP

* Age at data collection. AD: Autosomal dominant, AR: Autosomal recessive, AD/AR: Autosomal dominant & Autosomal recessive, XL: X-linked, PVS1: Very strong evidence of pathogenicity, PS3: Strong evidence of pathogenicity, PM1-PM6: Moderate strength evidence of pathogenicity, RP: Retinitis Pigmentosa, LCA: Leber Congenital Amaurosis.

Supplemental Table S6. Validating wild-type protein structures using different validation tools

Gene	***C-score***	***ProsA***	***ERRAT***	***VERIFY3D***
CRBA1	0.69	-3.78	-	30.77%
RPGRIP1	0.31	-6.97	89.9765	58.42%
GUCY2D	-2.81	-6.28	70.8395	67.68%
CFAP418	-2.6	-6.28	80.1047	42.51%

Each column in the table displays the score generated by a validation tool, providing an assessment of the overall quality of the protein model that was created.

Supplemental Table S7: The diagnostic yield of different genetic tests.

Genetic Test	***Utilization frequency/ per patients (n=49)***	***Solved cases (diagnostic yield)***	***Uncertain Cases***	***Unsolved Cases***
Whole Exome sequencing	37 (75.5%)	25 (67.6%) *p-value: 0.7314**	11 (29.8%)	1 (2.7%)
Gene Panel testing	8 (16.3%)	6 (75%) *p-value: 1.000**	0 (0%)	2 (25%)
Familial Targeted Testing	4 (8.2%)	3 (75%) *p-value: 1.000**	1 (25.0%)	0 (0.0%)
Total	49	34	12	3

* P-values were collected using the Fisher test to assess the genetic test diagnostic yield.

References
3. Khan AO. PHENOTYPE-GUIDED GENETIC TESTING OF PEDIATRIC INHERITED RETINAL DISEASE IN THE UNITED ARAB EMIRATES. Retina. 2020;40(9).
17. Sharon D, Ben-Yosef T, Goldenberg-Cohen N, Pras E, Gradstein L, Soudry S, et al. A nationwide genetic analysis of inherited retinal diseases in Israel as assessed by the Israeli inherited retinal disease consortium (IIRDC). Hum Mutat. 2020;41(1).
18. Jespersgaard C, Fang M, Bertelsen M, Dang X, Jensen H, Chen Y, et al. Molecular genetic analysis using targeted NGS analysis of 677 individuals with retinal dystrophy. Sci Rep. 2019;9(1).
19. Maltese PE, Colombo L, Martella S, Rossetti L, el Shamieh S, Sinibaldi L, et al. Genetics of Inherited Retinal Diseases in Understudied Ethnic Groups in Italian Hospitals. Front Genet. 2022 Jun 28;13.
20. Patel N, Alkuraya H, Alzahrani SS, Nowailaty SR, Seidahmed MZ, Alhemidan A, et al. Mutations in known disease genes account for the majority of autosomal recessive retinal dystrophies. Clin Genet. 2018;94(6).
21. Huang XF, Huang F, Wu KC, Wu J, Chen J, Pang CP, et al. Genotype-phenotype correlation and mutation spectrum in a large cohort of patients with inherited retinal dystrophy revealed by next-generation sequencing. Genetics in Medicine. 2015;17(4).
22. Khan AO, Alrashed M, Alkuraya FS. Clinical characterisation of the CABP4-related retinal phenotype. British Journal of Ophthalmology. 2013;97(3).
29. Maugeri A, Klevering BJ, Rohrschneider K, Blankenagel A, Brunner HG, Deutman AF, et al. Mutations in the ABCA4 (ABCR) gene are the major cause of autosomal recessive cone-rod dystrophy. Am J Hum Genet. 2000;67(4).
41. Patel N, Aldahmesh MA, Alkuraya H, Anazi S, Alsharif H, Khan AO, et al. Expanding the clinical, allelic, and locus heterogeneity of retinal dystrophies. Genetics in Medicine. 2016;18(6).
53 Schulz HL, Grassmann F, Kellner U, Spital G, Rüther K, Jägle H, et al. Mutation spectrum of the ABCA4 gene in 335 stargardt disease patients from a multicenter German cohort—impact of selected deep intronic variants and common SNPs. Invest Ophthalmol Vis Sci. 2017;58(1).
