# Supplementary material for: Exploring the Genetic Causes of Nonsyndromic Retinal Dystrophies in Qatar
Source: Genes (Basel). 2025 Nov 27;16(12):1415. doi: 10.3390/genes16121415 (PMC12733087; doi:10.3390/genes16121415)
Supplement: Supplementary file 1 [file genes-16-01415-s001.zip › Supp. Figures Final.pdf]

B

A. *PDE6B*: c.2407 A>G, p.Asn803Asp

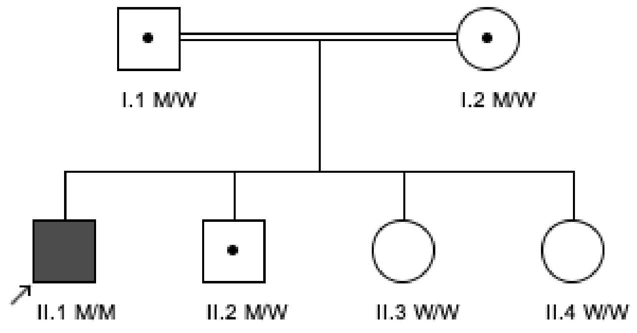

B. *ABCA4*: c.4753 C>T, p.Arg1585Trp

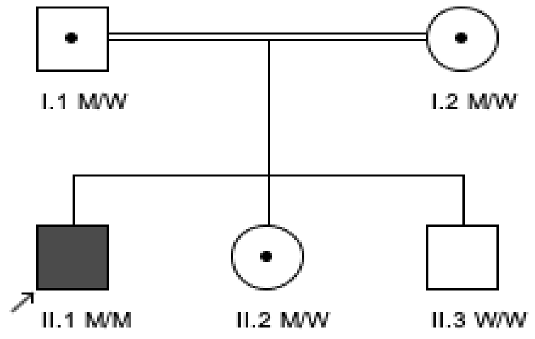

C. *PCDH15*: c.2897 G>C, p.Arg966Thr/ c.131 T>C, p.Val44Ala

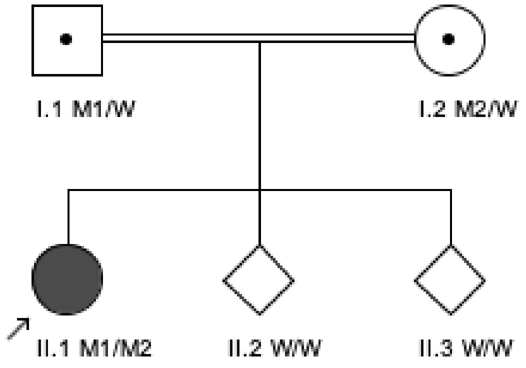

D. *GRM6*: c.281 G>C, p.Arg94Pro

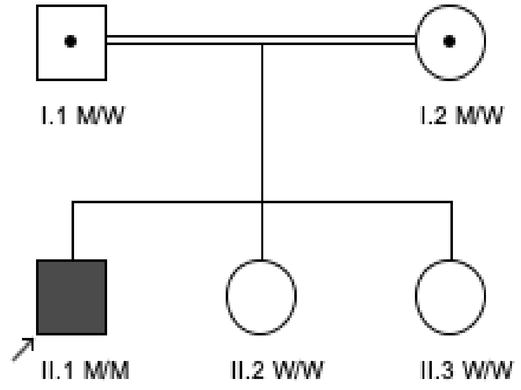

E. *CNM4*: c.509 T>C, Leu170Pro

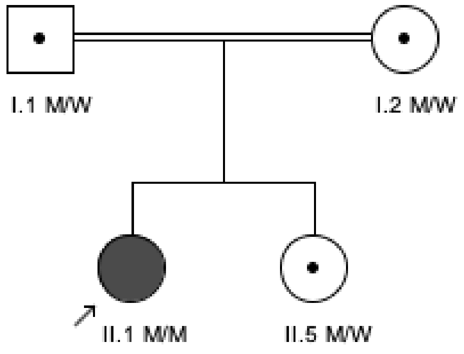

Supplemental Figure S1. Pedigrees showing variants segregating with the disease in cases re-evaluated as likely to be solved after family segregation studies. Generated by CeGaT pedigree chart designer. (W: wild type, M: mutant).

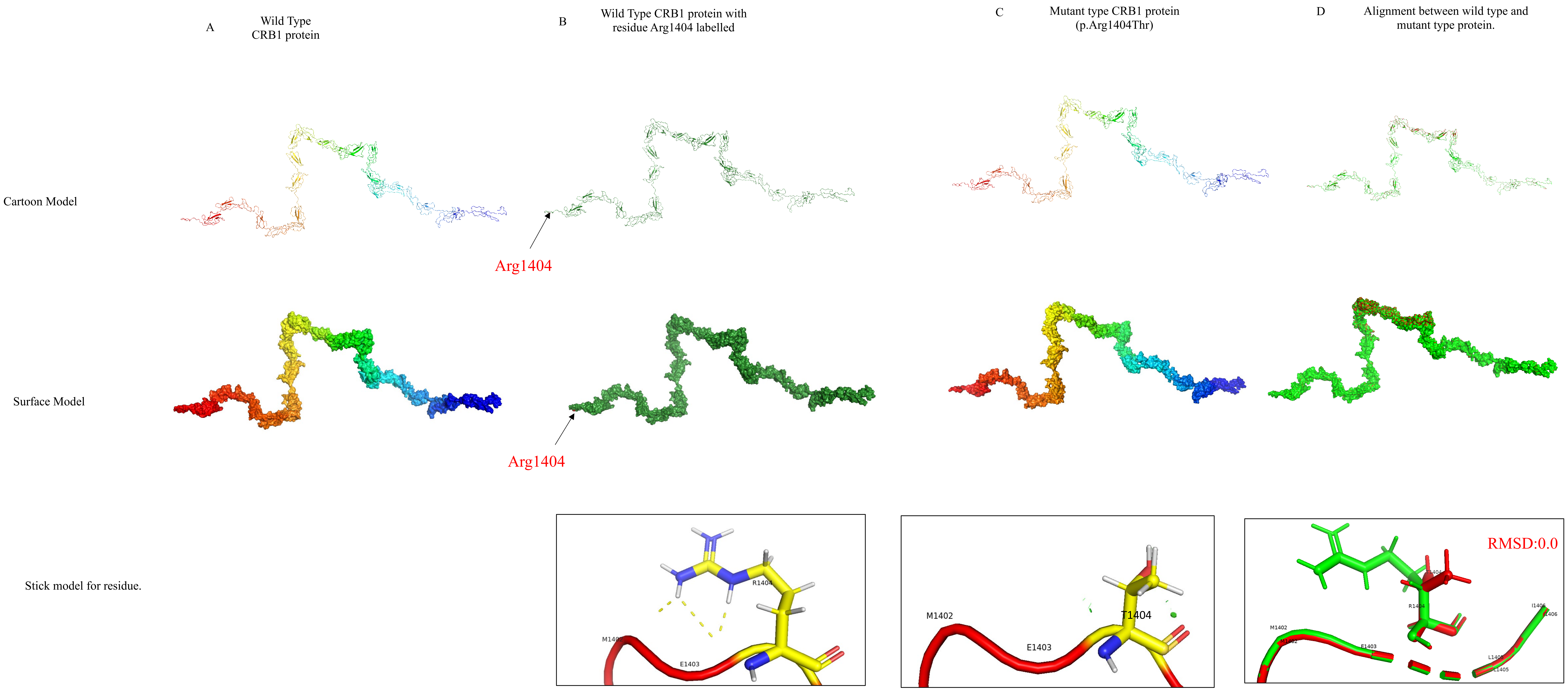

Supplemental Figure S2. *CRB1* predicted protein model using I-Tasser

A. CRB1 predicted protein model using I-Tasser (C score 0.69, Z score -3.78, ERRAT- no result, VERIFY3D 30.77%) visualized using PyMol as cartoon model and surface model. B residue Arg1404 is being labelled in the wild type predicted protein model, the stick model shows the residue Arg1404 have polar points to the residue E1403 (Glu1403) and M1402 (Met1402). C. the mutant type of protein model with that resulted by changing residue Arg1404 to Thr1404 showing in cartoon and surface model, the stick model for the new residue showing minor clashes and loss of polar interaction to E1403 (Glu1403) and M1402 (Met1402). D. alignment analysis between the wild type (green) and the mutant type (red) showing almost no misalignment in the overall protein structure (RMSD:0.0)

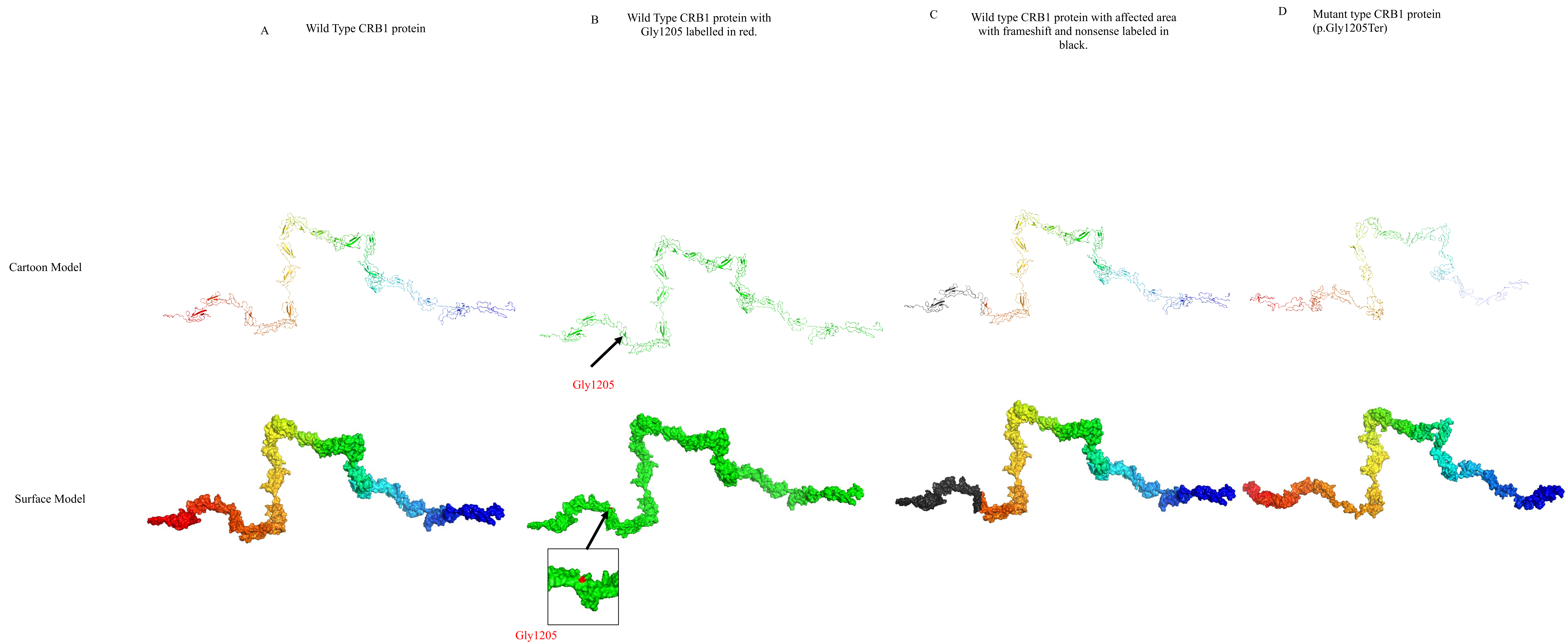

Supplemental Figure S3. *CRB1*(2) predicted protein model using I-Tasser

A. CRB1 predicted protein model using I-Tasser (C score 0.69, Z score -3.78, ERRAT-no result, VERIFY3D 30.77%) visualized using PyMol as cartoon model and surface model. B residue Gly1205 is being labelled in the wild type predicted protein model. C. The wild type CRB1 protein structure disruption caused by the nonsense mutation Met1205Ter is shown in black. D. the mutant type protein structure with the Gly1205 Ter in cartoon and surface model (RMSD = 63.693).

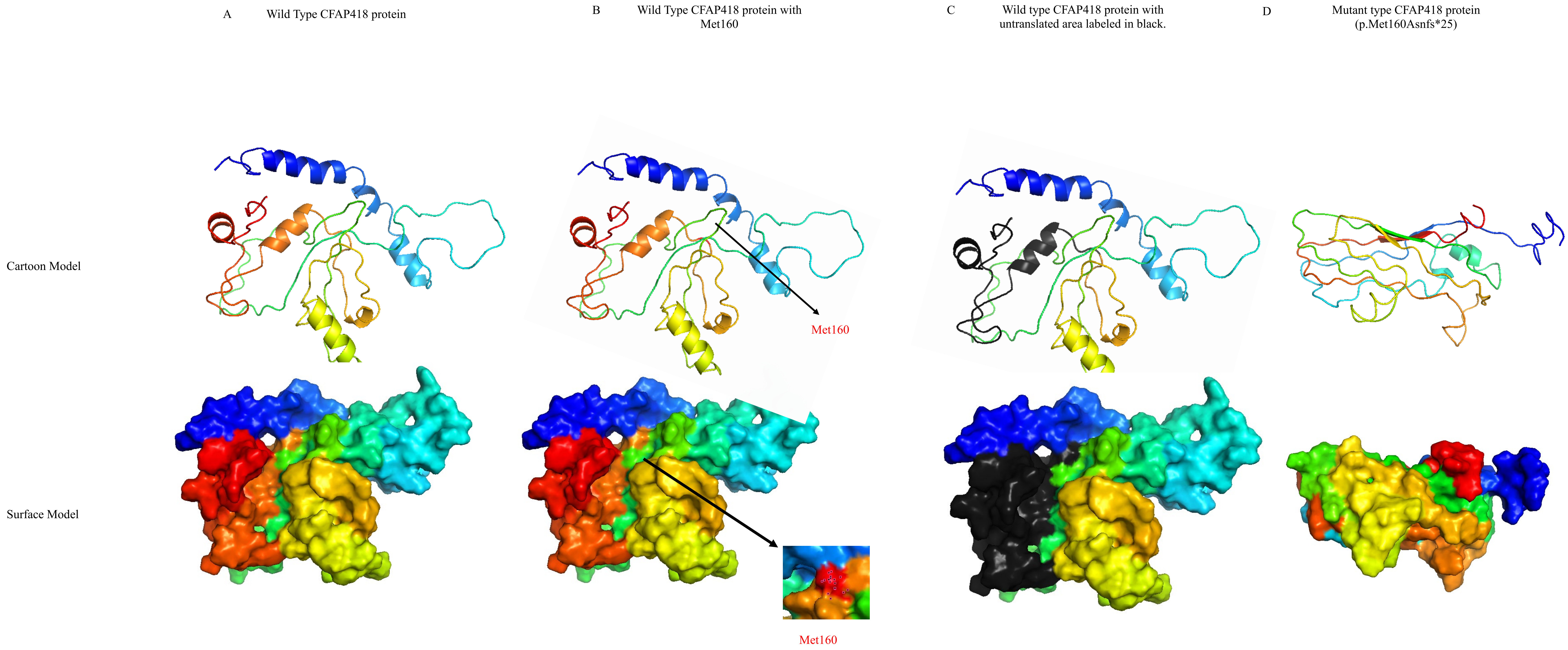

Supplemental Figure S4. *CFAP418* predicted protein model using I-Tasser

A. CFAP418 predicted protein model using I-Tasser (C score -2.6, Z score -6.28, ERRAT 80.1047, VERIFY3D 42.51%) visualized using PyMol as cartoon model and surface model. B residue Met160 is being labelled in the wild type predicted protein model. C. the wild type of protein model with the untranslated affected area labeled in black. D. the mutant type protein structure with the Met160Asnfs*fs in cartoon and surface model (RMSD = 22.119).

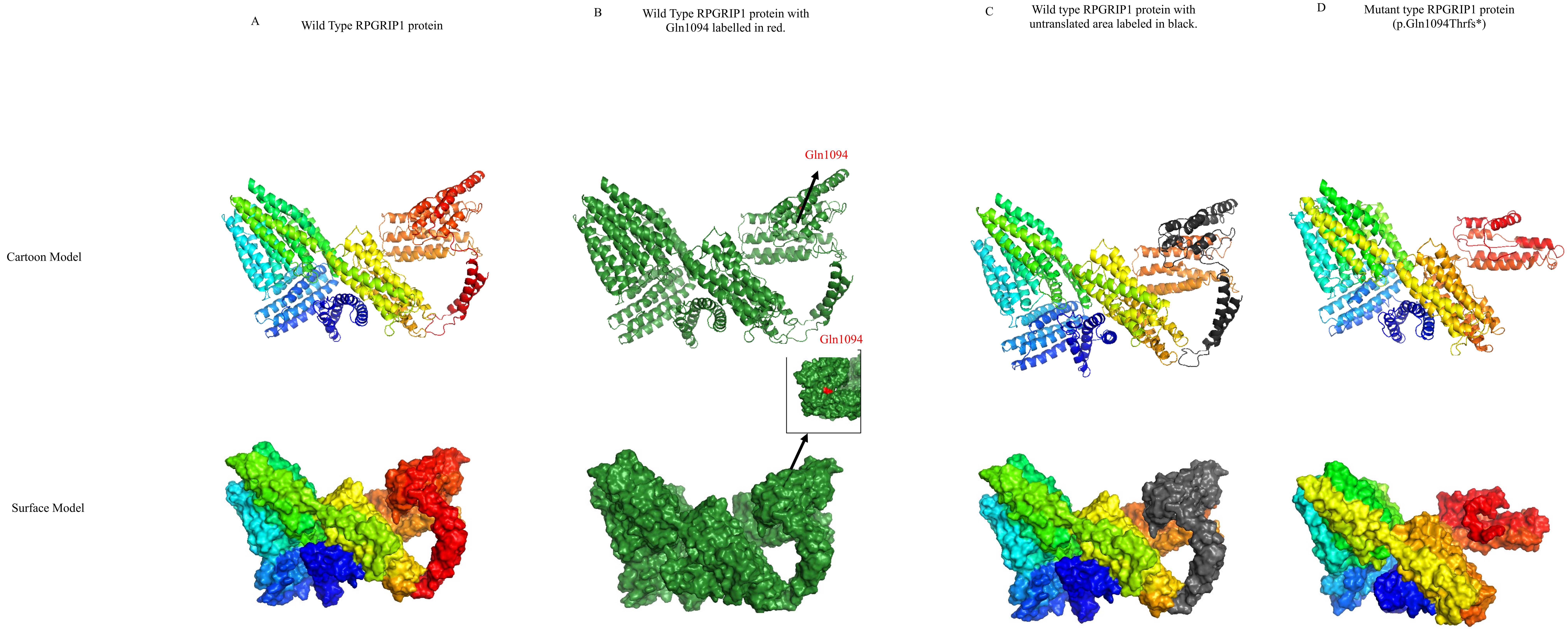

Supplemental Figure S5. *RPGRIP1* predicted protein model using I-Tasser

A. RPGRIP1 predicted protein model using I-Tasser (C score 0.31, Z score -6.97, ERRAT 89.9765, VERIFY3D 58.42%) visualized using PyMol as cartoon model and surface model. B residue Gln1094 is being labelled in the wild type. C. the wild type of protein model with the untranslated affected area labeled in black. D. the mutant type protein structure with the p.Gln1094Thrfs* in cartoon and surface model (RMSD = 1.047).

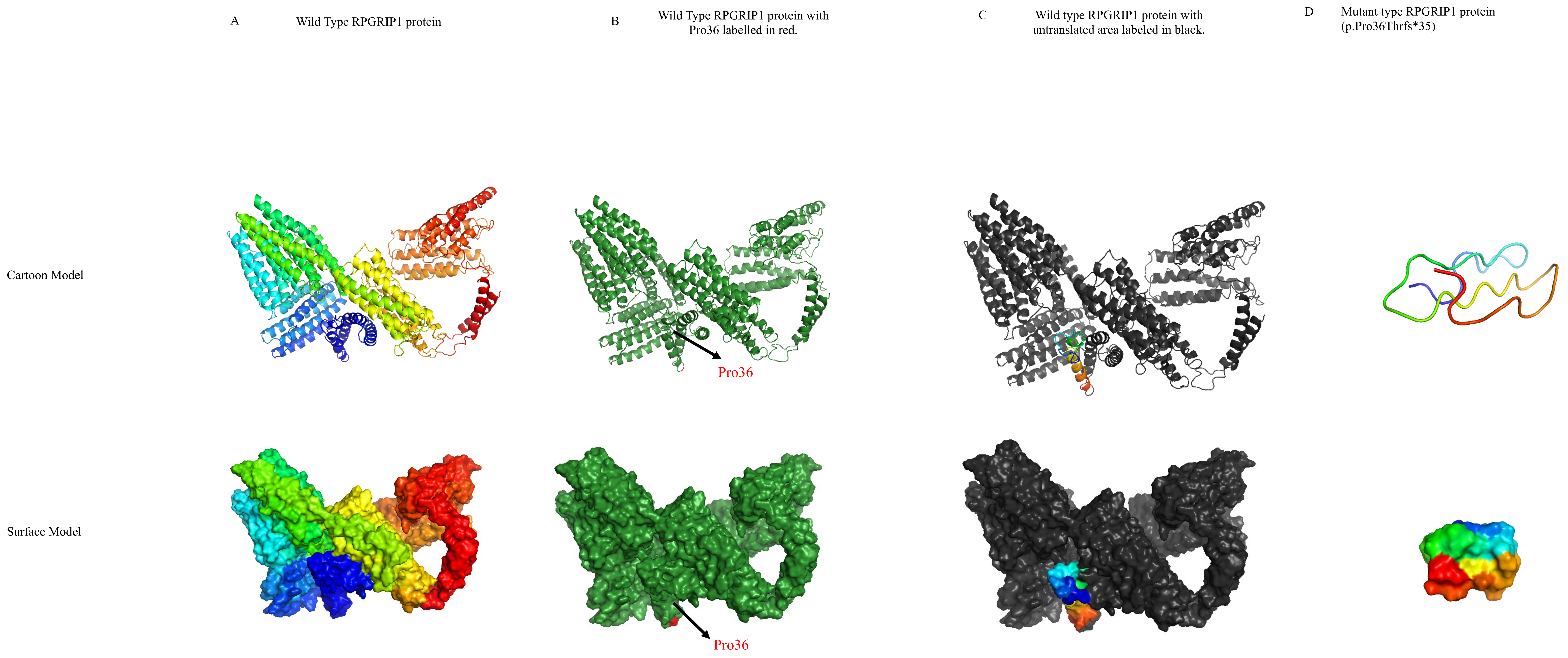

Supplemental Figure S6. *RPGRIP1*(2) predicted protein model using I-Tasser

A. RPGRIP1 predicted protein model using I-Tasser (C score 0.31, Z score -6.97, ERRAT 89.9765, VERIFY3D 58.42%) visualized using PyMol as cartoon model and surface model. B residue p.Pro36 is being labelled in the wild type. C. the wild type of protein model with the untranslated affected area labeled in black. D. the mutant type protein structure with the p.Pro36Thrfs*35 in cartoon and surface model (RMSD = 13.312).

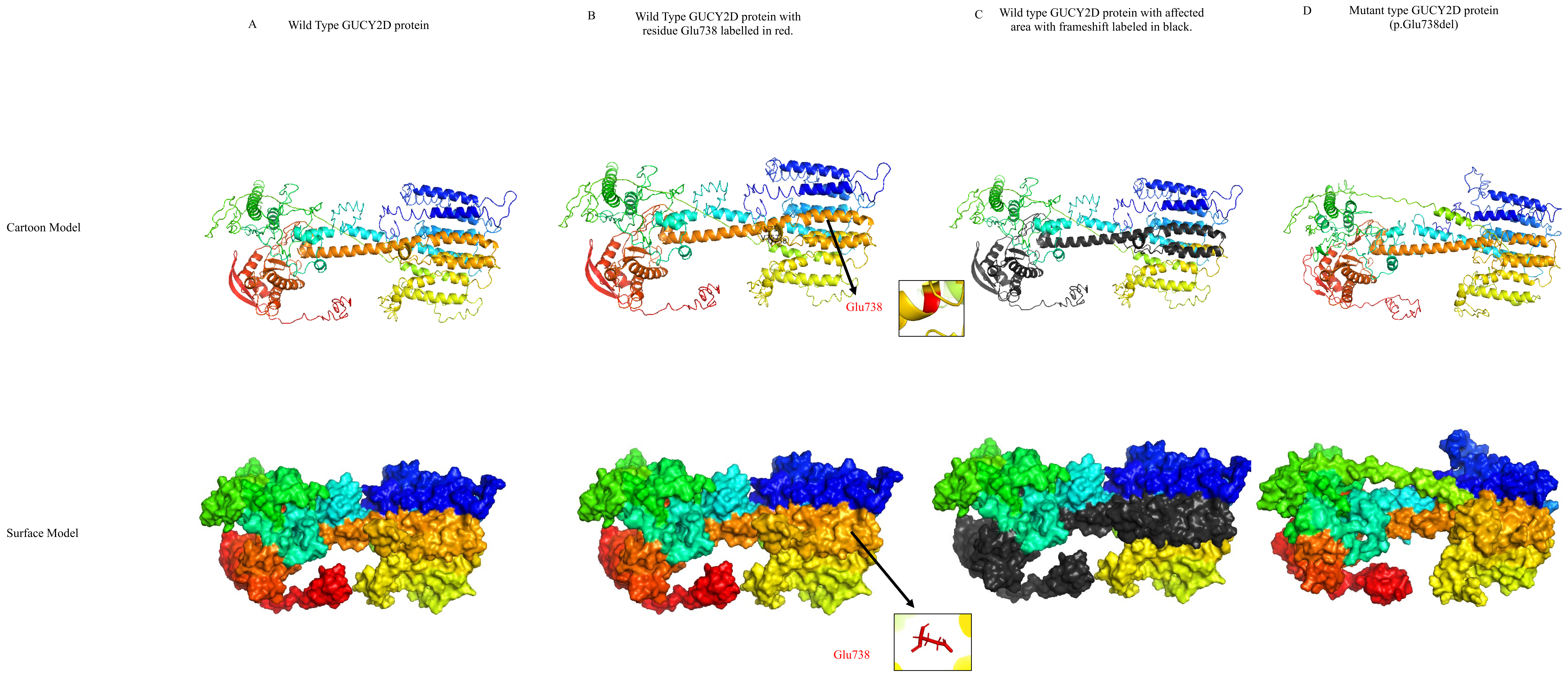
A
Wild Type GUCY2D protein
B
Wild Type GUCY2D protein with residue Glu738 labelled in red.
C
Wild type GUCY2D protein with affected area with frameshift labeled in black.
D
Mutant type GUCY2D protein (p.Glu738del)
Cartoon Model
Glu738
Surface Model
Glu738

E Alignment analysis between wild Type GUCY2D protein and mutant type Glu 738del

Cartoon Model

Surface Model

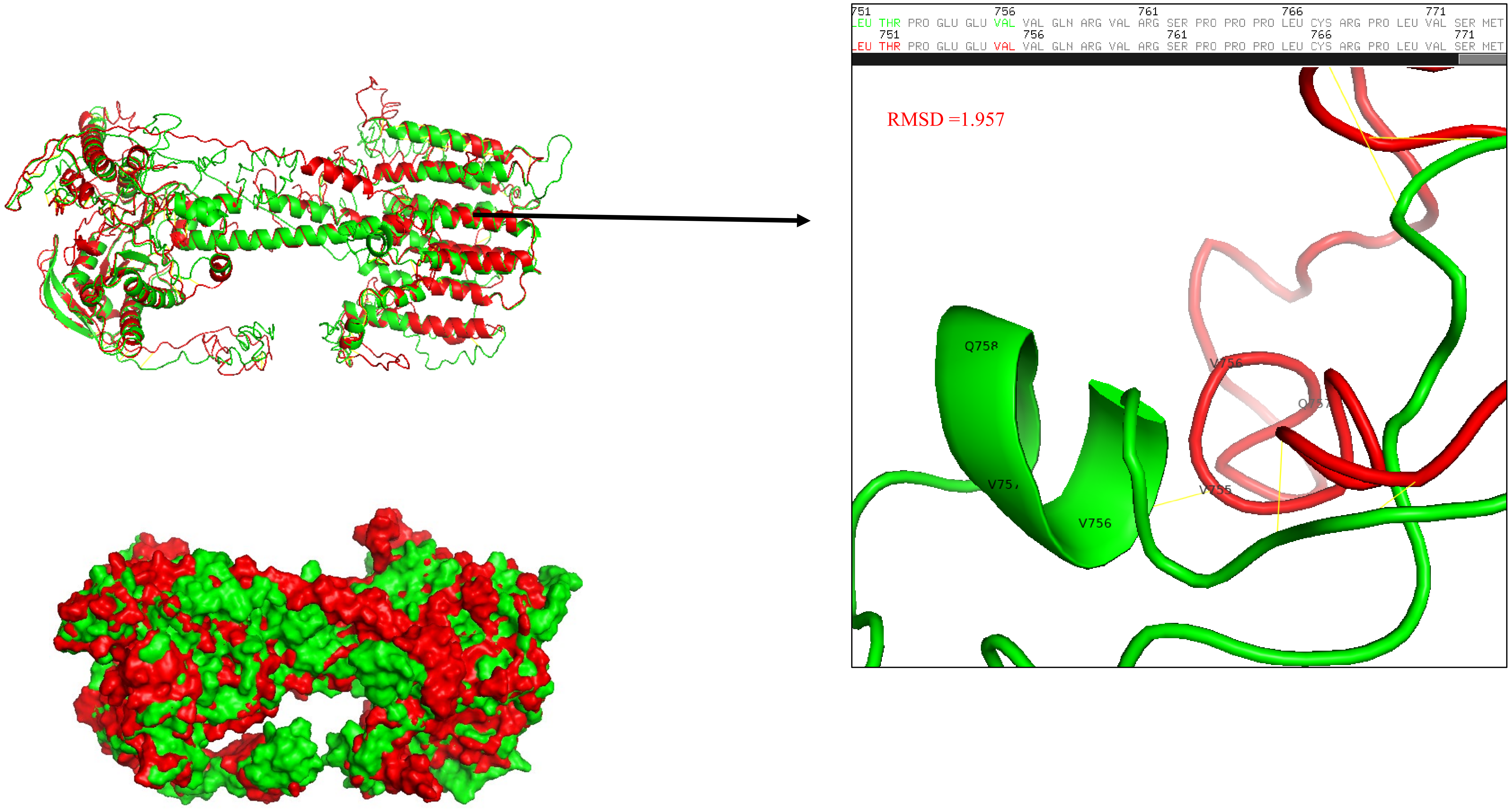

Supplemental Figure S7. *GUCY2D* predicted protein model using I-Tasser

A. GUCY2D predicted protein model using I-Tasser (C score -2.81, Z score -6.28, ERRAT 70.8395, VERIFY3D 67.68%) visualized using PyMol as cartoon model and surface model. B residue p.Glu738 is being labelled in the wild type. C. the wild type of protein model with the affected area with frameshift labeled in black. D. the mutant type protein structure with the p.Glu738del. E. alignment analysis between the wild type (green) and the mutant type (red) showing slight misalignment in the overall protein structure (RMSD:1.957)
